# Highly Emissive Hexa‐*peri*‐benzocoronene‐fluoranthene Hybrid as Easily Processable and Stable OLED Material

**DOI:** 10.1002/chem.202500742

**Published:** 2025-05-03

**Authors:** Alexander R. Krappe, Jacob C. Mayer, Wuai Zhang, Lina M. Filla, Giovanni Ligorio, Felix Hermerschmidt, Larissa S. Eitelhuber, Arne Güttler, Manuela Weber, Beate Paulus, Ute Resch‐Genger, Emil J. W. List‐Kratochvil, Siegfried Eigler

**Affiliations:** ^1^ Institut für Chemie und Biochemie (SupraFAB) Freie Universität Berlin Altensteinstr. 23a 14195 Berlin Germany; ^2^ Helmholtz‐Zentrum Berlin für Materialien und Energie GmbH Hahn‐Meitner‐Platz 1 14109 Berlin Germany; ^3^ Institut für Physik Institut für Chemie Humboldt‐Universität zu Berlin Zum Großen Windkanal 2 12489 Berlin Germany; ^4^ Center for the Science of Materials Berlin Zum Großen Windkanal 2 12489 Berlin Germany; ^5^ Institut für Chemie und Biochemie Freie Universität Berlin Arnimallee 22 14195 Berlin Germany; ^6^ Bundesanstalt für Materialforschung und ‐prüfung (BAM) Department 1 Division Biophotonics Richard‐Willstätter‐Straße 11 12489 Berlin Germany; ^7^ Institut für Chemie und Biochemie Freie Universität Berlin Fabeckstr. 34/36 14195 Berlin Germany

**Keywords:** carbon, dyes, emission, hydrocarbons, OLED

## Abstract

We report the synthesis of a fluorescent polycyclic aromatic hydrocarbon dye with a “symmetry‐broken” core, derived from the related hexa‐*peri*‐benzocoronene (HBC) core with a fluoranthene subunit. The fluorophore is composed of a pure carbon skeleton without heteroatoms and exhibits remarkable photoluminescence properties with a photoluminescence quantum yield (PLQY) of up to 67% in toluene, exceeding that of the parent HBC by a factor of 30. The single crystal X‐ray structure reveals the distorted polycyclic aromatic hydrocarbon structure, which is responsible for the optoelectronic properties, as supported by density functional theory calculations. We show that the new fluorescent dye can be readily used for the fabrication of organic light‐emitting diodes (OLED) without extensive optimization, whereby solubility in a variety of solvents and successful film formation are decisive.

## Graphical abstract

A “symmetry‐broken” hexabenzocoronene‐fluoranthene fluorescent dye was synthesized using an easily scalable reaction. Green emission at around 500 nm, a maximum photoluminescence quantum yield of 67% in toluene, and high solubility in a wide range of solvents result from the distorted structure. A green organic light‐emitting diode (OLED) was fabricated without extensive optimization showcasing easy processability of the molecule class.

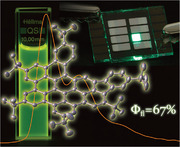

## 1 Introduction

Carbon has attracted considerable interest in scientific research over the last two decades and new chemical reaction principles were elaborated. In particular, the discovery of fullerenes, carbon nanotubes, and graphene opened up access to the chemistry of carbon‐rich compounds.^[^ ^1^, ^2^, ^3^ ^]^ Another line of development is currently focused on quasi‐0D particles, such as graphene quantum dots with their enormous potential for optoelectronic applications.^[^ ^4^ ^]^

In contrast to polydisperse materials, defined molecular structures enable a deeper understanding of the relationship between properties and chemical structures. In this context, early fundamental research on polycyclic aromatic hydrocarbons (PAHs) was conducted by Clar and Zander.^[^ ^5^, ^6^, ^7^ ^]^ The pioneering work of Müllen and colleagues on carbon‐rich molecules has led to the development of nanographenes.^[^ ^8^, ^9^, ^10^, ^11^ ^]^ Recent development is increasingly moving toward twisted or curved PAHs,^[^ ^12^, ^13^, ^14^, ^15^, ^16^ ^]^ with some resembling the hexa‐*peri*‐benzocoronene (HBC) motif.^[^ ^17^, ^18^, ^19^, ^20^, ^21^ ^]^ Applications have been shown for instance in fluorescence microscopy, field‐effect transistors, in conducting polymers, and bioimaging.^[^ ^22^, ^23^, ^24^ ^]^

In recent years, the development in the field of organic light‐emitting diodes (OLEDs) has made enormous progress evolving from first‐generation fluorescent OLEDs^[^ ^25^ ^]^ to more energy‐efficient but more unstable phosphorescent emitters (second generation).^[^ ^26^ ^]^ The need to become independent from heavy metals and rare earths led to the third generation of emitters based on thermally activated delayed fluorescence (TADF).^[^ ^27^ ^]^ Nevertheless, especially the fabrication of long‐term stable blue organic light emitters remains challenging and is still often realized using first‐generation systems.^[^ ^28^, ^29^ ^]^ To overcome solubility issues of condensed *π*‐systems nonplanar spiro systems have been developed that prevent dye aggregation,^[^ ^30^ ^]^ and to increase the emission efficiency many OLED materials make use of heteroatoms, i.e., B─N structural motifs or carbazole units.^[^ ^31^, ^32^, ^33^, ^34^, ^35^, ^36^ ^]^ In contrast, purely carbon‐based systems are rarely used,^[^ ^37^ ^]^ due to their large size,^[^ ^38^, ^39^, ^40^ ^]^ multi‐step synthesis, which is often not feasible on large scale, or poor solubility.^[^ ^41^ ^]^

Herein, we present a simple and easily scalable synthesis of a “symmetry‐broken” PAH dye, differing from the HBC structure by two carbon atoms, with a solubility of tens of mg·mL^−1^ and photoluminescence quantum yields (PLQY) exceeding 60%. In addition, it was possible to fabricate out‐of‐the‐box first‐generation OLEDs without extensive optimization.

## 2 Results and Discussion

**Figure 1:**
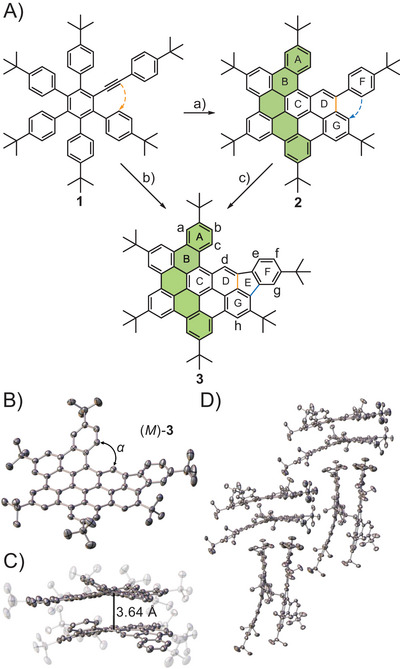
A) Synthetic pathway from pentaarylalkynylbenzene **1** to the compounds **2** and **3** in dependence of reaction conditions. a) 12 eq. FeCl_3_, 0 °C to rt, 16 hours, 80%; b) 18 eq. FeCl_3_, 0 °C to rt, 16 hours, 75% or 12 eq. DDQ, TfOH, −40 °C to 0 °C, 16 hours, 87%; c) 6 eq. FeCl_3_, 0 °C to rt, 16 hours. B) Structure of (*M*)‐**3** (left) in the solid state, *α* = 33.17°. Thermal ellipsoids shown at the 50% probability level, major part shown, hydrogen atoms, and solvent molecules omitted for clarity. C) Dimeric structure of compound **3** in the asymmetric unit. D) Sandwich herringbone‐type packing of **3** in the solid state.

Pentaarylalkynylbenzene **1** was prepared by a Diels‐Alder reaction between a cyclopentadienone and a dialkyne, as described previously,^[^ ^42^ ^]^ and then subsequently oxidized by means of a Scholl reaction^[^ ^43^, ^44^ ^]^ using iron(III) chloride as oxidant (Figure 1A).

Thereby, in addition to the planarization of the symmetrical polyphenylene system, the six‐membered ring D is initially formed by annulation of the alkyne. Based on this observation, compound **2** is obtained by slow addition of 12 equivalents of FeCl_3_, as studied earlier in our group.^[^ ^42^ ^]^ Although compound **2** differs from the parent hexakis‐*tert*‐butyl‐HBC (*t*‐Bu‐HBC) by only two carbon atoms introduced by an additional alkyne, a twisted structure with a nonplanar topology is formed. However, a new species, compound **3**, with a mass‐to‐charge ratio of *m/z *= 884 was detected using 18 equivalents FeCl_3_. The HBC‐fluoranthene hybrid **3**, which results from the C─C bond formation between rings G and F to form the five‐membered ring E, was isolated with a yield of 75% after separating unknown side products. The formation of five‐membered rings in the Scholl reaction was discovered by the group of Chi, 10 years ago.^[^ ^45^ ^]^ Further, we found that reacting **1** under Rathore's condition^[^ ^46^ ^]^ can suppress the formation of side products, increasing the yield of compound **3** to 87%. Even if more oxidant is used, no further ring closure is observed, since the only potential fusion between rings A and D would lead to high strain introduced by the curvature, which is energetically unfavorable. As determined by CHN combustion elemental analysis, compound **3** is yielded in >99% purity.

The formation of **3** could be verified by nuclear magnetic resonance (NMR) spectroscopy. The ^1^H signals of the bay region from rings A to G could be fully assigned using 2D NMR spectroscopy and nuclear overhauser effect (NOE) experiments (Supporting Information Chapter 11). The signal of the *tert*‐butyl group on ring G is shifted to lower field at 2.06 ppm and shows peak broadening due to the interaction with the spatially adjacent proton on ring F. In addition, the proton of ring D generates a highly low‐field shifted singlet signal at 9.93 ppm, which is 0.74 ppm at lower field than for the structurally related [4]helicene.^[^ ^47^ ^]^ This will probably result in an increased reactivity in this position.

Crystals of compound **3** suitable for single crystal X‐ray diffraction (Figure 1B) were grown via slow diffusion of methanol into a solution of toluene.^[^ ^48^ ^]^ Compound **3** crystallizes in a monoclinic lattice with the space group *P*2_1_/*n* in a sandwich herringbone‐type structure (Figure 1D) similar to the symmetric *t*‐Bu‐HBC.^[^ ^49^ ^]^ The asymmetric unit contains both (*P*)‐ and (*M*)‐enantiomer with an interplanar distance of 3.64 Å forming shell‐like dimers (Figure 1C and S3). The torsion angle between the rings A and D is *α* = 33.17° for both enantiomers and similar to the reported pentaphene **2** (32.46°).^[^ ^42^ ^]^ We argued earlier that the increase of curvature of the [4]helicene motif compared to the parent [4]helicene is due to the sterically demanding single benzene ring F in pentaphene **2**.^[^ ^42^ ^]^ Based on the almost identical racemization energies of **3** and pentaphene **2**, 11 and 10 kcal·mol^−1^, respectively, it is evident that phenyl ring F has almost no influence on the inversion barriers and curvature of the systems (Figure S22).

**Figure 2:**
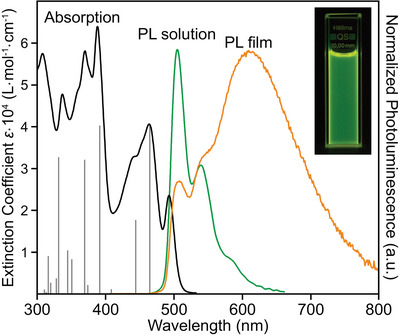
Absorption spectrum (black) with calculated oscillator strengths (gray) using TD‐DFT at the B3LYP/6‐311+G** level of theory, normalized photoluminescence (PL) spectrum in DCM (green) and in thin film (orange), picture of PL in solution shown as inset. Solution spectra measured at a concentration of 10^−5^ m. In the thin film, the spectrum is dominated by excimer emission.

Absorption and photoluminescence (PL) measurements of compound **3** were performed in protic (MeOH, EtOH) and aprotic solvents (acetone, THF, DCM, toluene, *n*‐hexane) of different polarity (Figures S6 and S7) and are exemplarily shown for DCM in Figure 2. Absorption and emission do not show any significant dependence on the solvent polarity (Table S1). The spectra are dominated by a large number of singlet electronic transitions which were assigned using time‐dependent DFT (TD‐DFT) (Table S3). The first excited state S_1_ is by 89% a HOMO‐LUMO transition with an oscillator strength of 0.53. Compound **3** shows two narrow PL bands at around 500 and 540 nm with a shoulder at 580 nm. In concentrated solutions (> 10^−3^ m), the formation of a red‐shifted excimer can be observed by color change in the emission from green to yellow (Figures S8 and S9). In the PL of the spin‐coated film, this leads to a broad emission above 600 nm. A similar behavior is commonly observed for molecules with large *π*‐systems.^[^ ^50^, ^51^, ^52^ ^]^

The molar extinction coefficient of compound **3** reaches a maximum of about 64,000 L·mol·cm^−1^ at 388 nm and is comparable to pentaphene **2**, whereas the absolutely determined PLQY is about three to six times higher, reaching a maximum of 67% in toluene in argon‐saturated solution (Table 1). Except for a few systems with a contorted pyrene or perylene backbone,^[^ ^40^, ^41^, ^53^ ^]^ this quantum yield is among the highest of reported PAH systems of similar size.^[^ ^54^, ^55^, ^56^, ^57^, ^58^, ^59^, ^60^, ^61^ ^]^

**Table 1:** Photoluminescence quantum yields (*Φ*), luminescence lifetimes (*τ*), and maximum wavelength of the first emission band *λ*_em_ of compound **3** in argon‐saturated solution. Polarity of the solvent classified using the Dimroth–Reichardt parameter $E_{N}^{T}$.^[^ ^62^ ^]^

	Solvent	*Φ* [%]	*τ* [ns]	*λ*_em_ [nm]
0.654	EtOH	61	9.2	500
0.309	DCM	51	5.6	505
0.099	Toluene	67	7.3	500
0.009	n‐Hexane	61	9.3	494
	Solid	4	4.8	596

The fluorescence lifetimes of 5.6 to 9.3 ns are considerably shorter than those of pentaphene **2** revealing lifetimes of 17.2 to 21.4 ns. This indicates that the electronic relaxations in compound **3** are more efficient than in compound **2**. The emission is quenched by the presence of oxygen, which is also accompanied by a shortening of the lifetimes (Figure S14). However, photostability experiments in air do not indicate degradation even for illumination at 300 nm (Figure S15). The increased PLQY of **3** compared with **2** is therefore mainly a result of rotational degrees of freedom due to the condensed ring F, reducing contributions from nonradiative decay processes, combined with a more efficient electronic relaxation pathway.

The emission of compound **3** in the solid state is red‐shifted with two maxima at 596 and 625 nm (Figure S10) compared to the emission of the dye solutions, while the PLQY of 4% is significantly lower than in solution as well as the fluorescence lifetime of 4.8 ns (Figure S16). The red‐shifted emission and the low fluorescence efficiency in the solid state are ascribed to the formation of excimers, which is also supported by the asymmetric unit in the crystal structure, consisting of two molecules in close proximity. However, in solution, and in particular in films, the formation of higher aggregates cannot be excluded.

**Figure 3:**
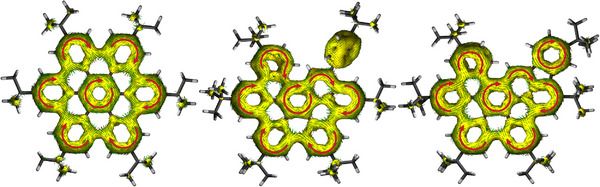
Anisotropy of the induced current density (AICD) plots of *t*‐Bu‐HBC (left), compound **2** (middle), and **3** (right) calculated at the B3LYP/def2‐TZVP level of theory, the selected *π*‐orbitals are listed in Table S4, contour level 0.026. Red arrows indicating diatropic ring current.

The nucleus‐independent chemical shift (NICS) values reveal a similar pattern for compounds **2** and **3** (Figure S23) in accordance with the most stable Clar resonance structure. The rings C exhibit the most diatropic value with NICS(1)_zz_ values of ‐29.6 and ‐27.5 for compounds **2** and **3**, respectively. Ring D experiences a certain aromatic stabilization with a value of ‐8.4, despite the expected isolated double bond according to the Clar resonance structure. The anisotropy of the induced current density (AICD) plots reveal a similar diatropic current of the *π*‐electrons between **2** and **3** above the polycyclic aromatic backbone also including the central ring C (Figure 3). In contrast to the symmetrical *t*‐Bu‐HBC, the central benzene ring is included in the overall ring current in the asymmetric structures **2** and **3** including also ring D as already determined by NICS analysis. Interestingly, in both structures ring F is practically not involved in the delocalization of the residual aromatic framework and should therefore have only a limited influence on the electronic properties of the molecules.

The electrochemical behavior of compound **3** was studied by cyclic voltammetry (Figures S17 and S18) to investigate the stability of **3** against reduction and oxidation. Differential pulse voltammetry (DPV) and square wave voltammetry (SWV) were used to detect the exact oxidation and reduction potentials (Figures S19 and S20). Compound **3** shows two distinct reversible one‐electron reductions at *E* ^0/‐I^_1/2_ = ‐1.96 V and *E* ^−I/‐II^_1/2_ = ‐2.34 V and one reversible one‐electron oxidation at *E* ^0/+I^_1/2_ = 0.60 V versus the ferrocene/ferrocenium (Fc/Fc^+^) redox couple. The broad range between oxidation and reduction made it necessary to measure the reduction processes in THF and the oxidation processes in DCM to avoid solvent degradation. At higher voltages, two further oxidations *E* ^+I/+II^_1/2_ = 0.92 V and *E* ^+II/+III^_1/2_ = 1.12 V can be observed. These oxidations are nonreversible, and result in the decomposition of the compound.

Compound **3**, which dissolves easily in chloroform at concentrations up to 10 mg·mL^−1^, readily form films upon evaporation of chlorinated solvents. Upon spin coating the chloroform solution, smooth, pinhole free films are formed. We hence investigated the possible fabrication of a first‐generation OLED and studied the optical and electronic properties of compound **3** in the film.

**Figure 4:**
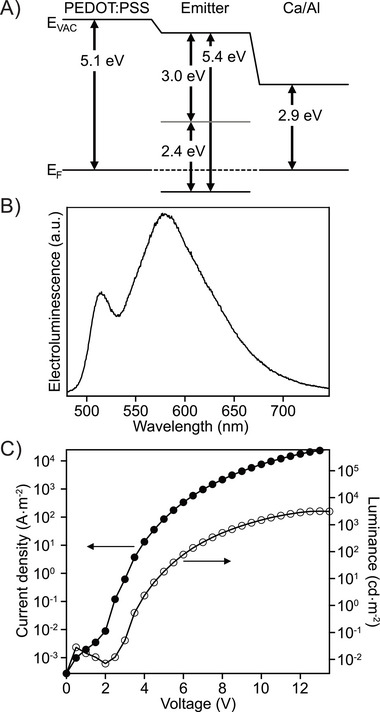
A) Energy diagram of the constructed OLED. B) Electroluminescence (EL) spectrum of the OLED. C) Current density‐voltage‐luminance (JVL) characteristics of the OLED, current density plotted with filled markers, luminance plotted with empty markers.

From the onset of the absorption in Figure 2, we determined the optical band gap *E*_OPT_ to 2.37 eV. Further, the energy levels of the molecule were determined via ultraviolet photoelectron spectroscopy (UPS) of the spin‐coated films (Figure S21). The HOMO (highest occupied molecular orbital) position was determined from the onset of the valence region in the UPS spectrum, indicating a HOMO level of 0.66 eV. Additionally, the Fermi level *E*_F_ was identified through the secondary electron cutoff (SECO) in the UPS spectrum, measured at 4.64 eV. A single‐layer device was fabricated, according to the procedure described in the Supporting Information (Chapter 9.1), to test the viability of compound **3** as an emitter material. The device structure is ITO/PEDOT:PSS/**3**/Ca/Al and the energy diagram of the device is shown in Figure 4A. The OLED shows a strong excimer emission at about 600 nm (Figure 4B), similar to the PL of the spin‐coated film. Current density‐voltage‐luminance (JVL) characteristics are shown in Figure 4C. The OLED has a turn‐on voltage (defined as the voltage where the luminance signal rises above the noise) of 3 V and a champion device reaches a luminance of 3458 cd·m^−2^, while the average luminance for the tested devices was at 3070 cd·m^−2^. The current starts to rise before the luminance, indicating nonideal charge carrier recombination.

**Figure 5:**
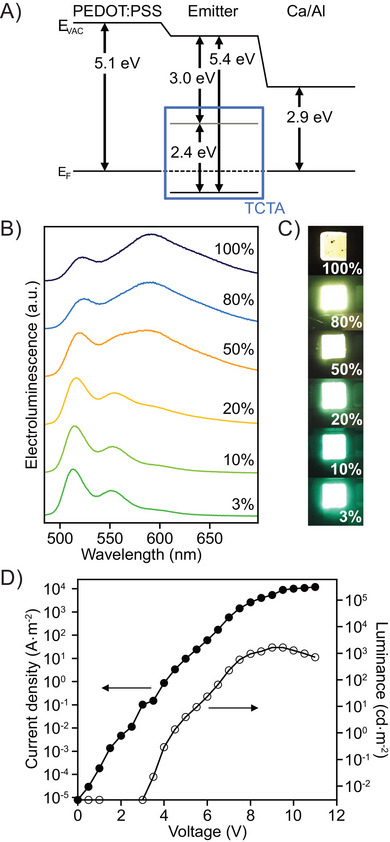
A) Energy diagram of the constructed OLED. B) EL spectra for OLEDs with different concentrations of emitter in the host. C) Photos of working devices. D) JVL characteristics of OLED with 3% emitter in the host, current density plotted with filled markers, luminance plotted with empty markers. JVL of OLEDs with other concentrations shown in Figure S24.

HBC and its derivatives are known to form aggregates through *π*‐*π* stacking.^[^ ^52^, ^63^ ^]^ As mentioned above, this behavior is observed for this molecule in pure films, which leads to excimer emission as can be seen in the PL of the thin film in Figure 2. A host material can be employed when producing OLEDs to prevent the formation of aggregates and avoid excimer emission. In this study, tris(4‐carbazoyl‐9‐ylphenyl)amine (TCTA), a widely used host material, was employed because it can easily be mixed with compound **3** in chloroform solutions and spin coated jointly.^[^ ^64^ ^]^ OLEDs with the structure ITO/PEDOT:PSS/**3** in TCTA (3‐100 wt%)/Ca/Al were constructed (Figure 5A). Figure 5B shows the emission spectra of the OLEDs with varying percentages of the emitter in the host material.

By varying the amount of the emitter molecule in the host, the emission spectrum of the OLED can be tuned to a desired color. At 3 wt% of the emitter in the host matrix, the excimer emission is fully suppressed and we only see the green monomer emission, similar to the PL of the molecule in solution. When the amount of the emitter molecules is increased to 20 wt%, excimer emission starts to appear and for higher amounts of emitter molecule in the host, the excimer emission exceeds the monomer emission. This also changes the overall color of the OLEDs from green to yellow, as can be seen from the photos in Figure 5C. The CIE coordinates shift from (0.26, 0.62) for 3 wt% to (0.48, 0.50) for a pure film of compound **3** (Figure S29).

The JVL characteristics for the OLED with 3 wt% emitter concentration are shown in Figure 5D. The turn‐on remains at 3 V and a champion device reached 1654 cd·m^−2^ with the average brightness of five devices at 1409 cd·m^−2^. The JVL data for other emitter concentrations are provided in the Supporting Information Figure S24. All devices showed a turn‐on voltage of around 3 V and reached luminances between 840 cd·m^−2^ and 2370 cd·m^−2^. Since the charge carrier recombination mechanism varies depending on the presence of a host material, and because the luminance measurement in candela depends on the wavelength of the emitted light, no clear trend in device brightness can be correlated to the emitter concentration in the host. The current efficacies were 0.46 cd·A^−1^ for the OLED with 3 wt% **3** in the TCTA host, and 0.25 cd·A^−1^ for the OLED with a pure film of compound **3**. The current efficacies plateau between 5 and 8 V and we see a small roll‐off above 10 V (Figure S25). To improve the charge carrier recombination and performance of the OLEDs, devices with the electron blocking layer poly(9,9‐dioctylfluorene‐*alt*‐*N*‐(4‐*sec*‐butylphenyl)‐diphenyl‐amine) (TFB) and hole blocking layer 1,3,5‐tri(*m*‐pyridin‐3‐ylphenyl)benzene (TmPyPB) were fabricated. While the overall luminance of the OLEDs did not increase, the current efficacy increased to 1.47 cd·A^−1^ for the OLED with a host material, while showing no improvement for the OLEDs without a host material. A summary of the device parameters including the luminous power efficiency can be found in the Supporting Information Table S5.

## 3 Conclusion

Unlike the symmetric HBC system, which results from six oxidative C─C bond formations, the “symmetry‐broken” system features six alternative oxidative C─C bond formations, including the formation of a five‐membered ring, which is caused by two additional carbon atoms introduced in the synthesis through a dialkyne. This results in a PAH fluorescent dye with improved solubility and photophysical properties. The combination of *tert*‐butyl groups and the distortion of the *π*‐system are essential for the high solubility and therefore the ease of fabrication of stable first‐generation OLEDs under air with minimal optimization. The higher emission efficiency of compound **3** compared to compound **2** is attributed to its reduced conformational degrees of freedom and shorter lifetimes, which, supported by computational studies, suggest a more efficient emissive electronic relaxation pathway. The use of compound **3** together with a host material enables control over excimer emission, allowing for color tuning of the device.

## Supporting Information

The authors have cited additional references within the Supporting Information.^[^ ^65^, ^66^, ^67^, ^68^, ^69^, ^70^, ^71^, ^72^, ^73^, ^74^, ^75^, ^76^, ^77^, ^78^, ^79^, ^80^, ^81^, ^82^, ^83^, ^84^, ^85^, ^86^, ^87^, ^88^, ^89^, ^90^ ^]^ Raw data are provided under http://doi.org/10.17169/refubium‐46092.

## Conflict of Interests

The authors declare no conflict of interest.

## Supporting information



Supporting Information

## Data Availability

The data that support the findings of this study are openly available in refubium at https://doi.org/10.17169/refubium‐46092, reference number 46092.

## References

[1] X. Ye , M. Qi , M. Chen , L. Zhang , J. Zhang , Adv. Mater. Interfaces 2023, 10, 2201941.

[2] A. Hirsch , Angew. Chem. Int. Ed. 1993, 32, 1138.

[3] S. Eigler , A. Hirsch , Angew. Chem. Int. Ed. 2014, 53, 7720. 10.1002/anie.201402780 24962439

[4] F. Yuan , T. Yuan , L. Sui , Z. Wang , Z. Xi , Y. Li , X. Li , L. Fan , Z. Tan , A. Chen , M. Jin , S. Yang , Nat. Commun. 2018, 9, 2249. 29884873 10.1038/s41467-018-04635-5 PMC5993800

[5] E. Clar , M. Zander , J. Chem. Soc. 1957, 4616.

[6] E. Clar , C. T. Ironside , M. Zander , Tetrahedron 1966, 22, 3527.

[7] E. Clar , The Aromatic Sextet, John Wiley & Sons, London, New York, Sidney, Toronto, 1972.

[8] F. Dötz , J. D. Brand , S. Ito , L. Gherghel , K. Müllen , J. Am. Chem. Soc. 2000, 122, 7707.

[9] C. D. Simpson , J. D. Brand , A. J. Berresheim , L. Przybilla , H. J. Räder , K. Müllen , Chem. Eur. J. 2002, 8, 1424. 11921226 10.1002/1521-3765(20020315)8:6<1424::aid-chem1424>3.0.co;2-z

[10] Y. Hu , X.‐Y. Wang , P.‐X. Peng , X.‐C. Wang , X.‐Y. Cao , X. Feng , K. Müllen , A. Narita , Angew. Chem. Int. Ed. 2017, 56, 3374. 10.1002/anie.201610434 27966818

[11] A. Narita , X.‐Y. Wang , X. Feng , K. Müllen , Chem. Soc. Rev. 2015, 44, 6616. 26186682 10.1039/c5cs00183h

[12] K. Baumgärtner , A. L. Meza Chincha , A. Dreuw , F. Rominger , M. Mastalerz , Angew. Chem. Int. Ed. 2016, 55, 15594. 10.1002/anie.201607740 27649907

[13] Y. Zhu , Z. Xia , Z. Cai , Z. Yuan , N. Jiang , T. Li , Y. Wang , X. Guo , Z. Li , S. Ma , D. Zhong , Y. Li , J. Wang , J. Am. Chem. Soc. 2018, 140, 4222. 29537262 10.1021/jacs.8b01447

[14] X.‐J. Zhao , Y.‐Y. Ju , Y.‐M. Su , C. Tang , Q. Zheng , L. Feng , C. Wang , K. Müllen , Y.‐Z. Tan , J. Am. Chem. Soc. 2023, 145, 19333. 37638550 10.1021/jacs.3c05662

[15] S. H. Pun , K. M. Cheung , H. Chen , Y. Wang , Q. Miao , Angew. Chem. Int. Ed. 2022, 61, e202113203. 10.1002/anie.202113203 34921485

[16] For further references on recent advances in nanographene chemistry please refer to: H. V. Anderson , N. D. Gois , W. A. Chalifoux , Org. Chem. Front. 2023, 10, 4167.

[17] D. Reger , P. Haines , F. W. Heinemann , D. M. Guldi , N. Jux , Angew. Chem. Int. Ed. 2018, 57, 5938. 10.1002/anie.201800585 29508521

[18] P. Izquierdo‐García , J. M. Fernández‐García , S. M. Rivero , M. Šámal , J. Rybáček , L. Bednárová , S. Ramírez‐Barroso , F. J. Ramírez , R. Rodríguez , J. Perles , D. García‐Fresnadillo , J. Crassous , J. Casado , I. G. Stará , N. Martín , J. Am. Chem. Soc. 2023, 145, 11599. 37129470 10.1021/jacs.3c01088 PMC10236438

[19] P. Izquierdo‐García , J. M. Fernández‐García , J. Perles , N. Martín , J. Am. Chem. Soc. 2024, 146, 34943. 39642941 10.1021/jacs.4c14544 PMC11664500

[20] M. Buendía , J. M. Fernández‐García , J. Perles , S. Filippone , N. Martín , Nat. Synth. 2024, 3, 545.

[21] For further references on chiral HBC‐based nanographenes please refer to: R. Li , D. Wang , P. An , Beilstein J. Org. Chem. 2023, 19, 736. 37284588 10.3762/bjoc.19.54 PMC10241098

[22] Z. Liu , H. Qiu , C. Wang , Z. Chen , B. Zyska , A. Narita , A. Ciesielski , S. Hecht , L. Chi , K. Müllen , P. Samorì , Adv. Mater. 2020, 32, 2001268. 10.1002/adma.202001268 32378243

[23] X. Liu , S.‐Y. Chen , Q. Chen , X. Yao , M. Gelléri , S. Ritz , S. Kumar , C. Cremer , K. Landfester , K. Müllen , S. H. Parekh , A. Narita , M. Bonn , Angew. Chem. Int. Ed. 2020, 59, 496. 10.1002/anie.201909220 PMC6972658 31657497

[24] Y. Gu , Z. Qiu , K. Müllen , J. Am. Chem. Soc. 2022, 144, 11499. 35671225 10.1021/jacs.2c02491 PMC9264366

[25] C. W. Tang , S. A. VanSlyke , Appl. Phys. Lett. 1987, 51, 913.

[26] M. A. Baldo , D. F. O'Brien , Y. You , A. Shioustikov , S. Sibley , M. E. Thompson , S. R. Forrest , Nature 1998, 395, 151.

[27] H. Uoyama , K. Goushi , K. Shizu , H. Nomura , C. Adachi , Nature 2012, 492, 234. 23235877 10.1038/nature11687

[28] J.‐H. Lee , C.‐H. Chen , P.‐H. Lee , H.‐Y. Lin , M.‐K. Leung , T.‐L. Chiu , C.‐F. Lin , J. Mater. Chem. C 2019, 7, 5874.

[29] E. Tankelevičiūtė , I. D. W. Samuel , E. Zysman‐Colman , J. Phys. Chem. Lett. 2024, 15, 1034. 38259039 10.1021/acs.jpclett.3c03317 PMC10839906

[30] Y. Han , L. Bai , J. Lin , X. Ding , L. Xie , W. Huang , Adv. Funct. Mater. 2021, 31, 2105092.

[31] Z. Sun , C. Yi , Q. Liang , C. Bingi , W. Zhu , P. Qiang , D. Wu , F. Zhang , Org. Lett. 2020, 22, 209. 31860317 10.1021/acs.orglett.9b04167

[32] F. Zhang , V. Brancaccio , F. Saal , U. Deori , K. Radacki , H. Braunschweig , P. Rajamalli , P. Ravat , J. Am. Chem. Soc. 2024, 146, 29782. 39435966 10.1021/jacs.4c11404

[33] H. Chen , M. Du , C. Qu , Q. Jin , Z. Tao , R. Ji , G. Zhao , T. Zhou , Y. Lou , Y. Sun , W. Jiang , L. Duan , Y. Zhang , Angew. Chem. Int. Ed. 2024, 64, e202415400. 10.1002/anie.202415400 39258563

[34] S. Oner , M. R. Bryce , Mater. Chem. Front. 2023, 7, 4304.

[35] T. Feng , X. Nie , D. Liu , L. Wu , C. Y. Liu , X. Mu , Z. Xin , B. Liu , H. Qi , J. Zhang , W. Li , S.‐J. Su , Z. Ge , Angew. Chem. Int. Ed. 2024, 64, e202415113. 10.1002/anie.202415113 39297652

[36] J. Wang , D. Chen , J. M. Moreno‐Naranjo , F. Zinna , L. Frédéric , D. B. Cordes , A. P. McKay , M. J. Fuchter , X. Zhang , E. Zysman‐Colman , Chem. Sci. 2024, 15, 16917. 39328198 10.1039/d4sc03478c PMC11420764

[37] C.‐L. Wu , C.‐H. Chang , Y.‐T. Chang , C.‐T. Chen , C.‐T. Chen , C.‐J. Su , J. Mater. Chem. C 2014, 2, 7188.

[38] S. Ma , J. Gu , C. Lin , Z. Luo , Y. Zhu , J. Wang , J. Am. Chem. Soc. 2020, 142, 16887. 32900184 10.1021/jacs.0c08555

[39] S. Castro‐Fernández , C. M. Cruz , I. F. A. Mariz , I. R. Márquez , V. G. Jiménez , L. Palomino‐Ruiz , J. M. Cuerva , E. Maçôas , A. G. Campaña , Angew. Chem. Int. Ed. 2020, 59, 7139. 10.1002/anie.202000105 32159924

[40] G. F. Huo , W.‐T. Xu , J. Hu , Y. Han , W. Fan , W. Wang , Z. Sun , H.‐B. Yang , J. Wu , Angew. Chem. Int. Ed. 2025, 64, e202416707. 10.1002/anie.202416707 39363697

[41] C. Wallerius , O. Erdene‐Ochir , E. V. Doeselar , R. Alle , A. T. Nguyen , M. F. Schumacher , A. Lützen , K. Meerholz , S. H. Pun , Precis. Chem. 2024, 2, 488. 39474393 10.1021/prechem.4c00038 PMC11501045

[42] P. Rietsch , J. Soyka , S. Brülls , J. Er , K. Hoffmann , J. Beerhues , B. Sarkar , U. Resch‐Genger , S. Eigler , Chem. Commun. 2019, 55, 10515. 10.1039/c9cc05451k 31414103

[43] R. Scholl , C. Seer , Justus Liebigs Ann. Chem. 1912, 394, 111.

[44] R. Scholl , C. Seer , R. Weitzenböck , Ber. Dtsch. Chem. Ges. 1910, 43, 2202.

[45] A. N. Lakshminarayana , J. Chang , J. Luo , B. Zheng , K.‐W. Huang , C. Chi , Chem. Commun. 2015, 51, 3604. 10.1039/c4cc09812a 25634022

[46] L. Zhai , R. Shukla , R. Rathore , Org. Lett. 2009, 11, 3474. 19594139 10.1021/ol901331p

[47] S. Paul , R. Jana , J. Ray , Synlett 2010, 10, 1463.

[48] Deposition numbers 2401868 (for **1**), and 2401878 (for **3**) contain the supplementary crystallographic data for this paper. These data are provided free of charge by the joint Cambridge Crystallographic Data Centre and Fachinformationszentrum Karlsruhe Access Structures service.

[49] G. R. Desiraju , A. Gavezzotti , Acta Crystallogr. Sect. B: Struct. Sci. Cryst. Eng. Mater. 1989, 45, 473.

[50] V. J. Chebny , C. Gwengo , J. R. Gardinier , R. Rathore , Tetrahedron Lett. 2008, 49, 4869.

[51] J. R. Lakowicz , Principles of Fluorescence Spectroscopy, 3rd ed., Springer, New York, 2006.

[52] S. Setia , S. K. Pal , ChemistrySelect 2016, 5, 880.

[53] J.‐K. Li , X.‐Y. Chen , W.‐L. Zhao , Y.‐L. Guo , Y. Zhang , X.‐C. Wang , A. C.‐H. Sue , X.‐Y. Cao , M. Li , C.‐F. Chen , X.‐Y. Wang , Angew. Chem. Int. Ed. 2023, 62, e202215367. 10.1002/anie.202215367 36428269

[54] Z. Qiu , C.‐W. Ju , L. Frédéric , Y. Hu , D. Schollmeyer , G. Pieters , K. Müllen , A. Narita , J. Am. Chem. Soc. 2021, 143, 4661. 33735570 10.1021/jacs.0c13197 PMC8041289

[55] X. Xiao , S. K. Pedersen , D. Aranda , J. Yang , R. A. Wiscons , M. Pittelkow , M. L. Steigerwald , F. Santoro , N. J. Schuster , C. Nuckolls , J. Am. Chem. Soc. 2021, 143, 983. 33377771 10.1021/jacs.0c11260

[56] F. Morita , J. Nogami , A. J. Araujo Dias , S. Kinoshita , Y. Nagashima , K. Tanaka , Eur. J. Org. Chem. 2022, 2022, e202200690.

[57] R. Yamano , Y. Shibata , K. Tanaka , Chem.Eur. J. 2018, 24, 6364. 29349825 10.1002/chem.201706008

[58] H.‐C. Huang , Y.‐C. Hsieh , P.‐L. Lee , C.‐C. Lin , Y.‐S. Ho , W.‐K. Shao , C.‐T. Hsieh , M.‐J. Cheng , Y.‐T. Wu , J. Am. Chem. Soc. 2023, 145, 10304. 37099267 10.1021/jacs.3c01647

[59] J. Borstelmann , L. Schneider , F. Rominger , F. Deschler , M. Kivala , Angew. Chem. Int. Ed. 2024, 63, e202405570. 10.1002/anie.202405570 38716767

[60] M. A. Medel , R. Tapia , V. Blanco , D. Miguel , S. P. Morcillo , A. G. Campaña , Angew. Chem. Int. Ed. 2021, 60, 6094. 10.1002/anie.202015368 33337575

[61] J. M. Fernández‐García , P. J. Evans , S. M. Rivero , I. Fernández , D. García‐Fresnadillo , J. Perles , J. Casado , N. Martín , J. Am. Chem. Soc. 2018, 140, 17188. 30431273 10.1021/jacs.8b09992

[62] C. Reichardt , Chem. Rev. 1994, 94, 2319.

[63] D. Wasserfallen , I. Fischbach , N. Chebotareva , M. Kastler , W. Pisula , F. Jäckel , M. D. Watson , I. Schnell , J. P. Rabe , H. W. Spiess , K. Müllen , Adv. Funct. Mater. 2005, 15, 1585.

[64] Y. Tao , C. Yang , J. Qin , Chem. Soc. Rev. 2011, 40, 2943. 21369622 10.1039/c0cs00160k

[65] M. Götze , L. Polewski , L. Bechtella , K. Pagel , J. Am. Soc. Mass Spectrom. 2023, 34, 2403. 37602654 10.1021/jasms.3c00214 PMC10557379

[66] O. V. Dolomanov , L. J. Bourhis , R. J. Gildea , J. A. K. Howard , H. Puschmann , J. Appl. Crystallogr. 2009, 42, 339. 10.1107/S0021889811041161 PMC3236671 22199401

[67] A. L. Spek , J. Appl. Crystallogr. 2003, 36, 7.

[68] G. M. Sheldrick , Acta Crystallogr. Sect. C: Struct. Chem. 2015, 71, 3. 25567568 10.1107/S2053229614024218 PMC4294323

[69] U. T. Mueller‐Westerhoff , M. Zhou , J. Org. Chem. 1994, 59, 4988.

[70] S. K. Sadhukhan , C. Viala , A. Gourdon , Synthesis 2003, 10, 1521.

[71] X. Geng , J. T. Mague , J. P. Donahue , R. A. Pascal , J. Org. Chem. 2016, 81, 3838. 27040596 10.1021/acs.joc.6b00492

[72] J. R. Johnson , O. Grummitt , Org. Synth. 1943, 23, 92.

[73] G. Cheng , H. Zhang , X. Cui , RSC Adv. 2014, 4, 1849.

[74] F. Neese , Wiley Interdiscip. Rev. Comput. Mol. Sci. 1606, 2022, 12e.

[75] M. J. Frisch , G. W. Trucks , H. B. Schlegel , G. E. Scuseria , M. A. Robb , J. R. Cheeseman , G. Scalmani , V. Barone , G. A. Petersson , H. Nakatsuji , X. Li , M. Caricato , A. V. Marenich , J. Bloino , B. G. Janesko , R. Gomperts , B. Mennucci , H. P. Hratchian , J. V. Ortiz , A. F. Izmaylov , J. L. Sonnenberg , Williams , F. Ding , F. Lipparini , F. Egidi , J. Goings , B. Peng , A. Petrone , T. Henderson , D. Ranasinghe , et al., Gaussian 16, Revision C.01, Gaussian, Inc, Wallingford, CT 2016.

[76] J.‐D. Chai , M. Head‐Gordon , Phys. Chem. Chem. Phys. 2008, 10, 6615. 18989472 10.1039/b810189b

[77] F. Weigend , R. Ahlrichs , Phys. Chem. Chem. Phys. 2005, 7, 3297. 16240044 10.1039/b508541a

[78] S. Grimme , J. Antony , S. Ehrlich , H. Krieg , J. Chem. Phys. 2010, 132, 154104. 20423165 10.1063/1.3382344

[79] S. Grimme , S. Ehrlich , L. Goerigk , J. Comput. Chem. 2011, 32, 1456. 21370243 10.1002/jcc.21759

[80] A. D. Becke , J. Chem. Phys. 1993, 98, 5648.

[81] C. Lee , W. Yang , R. G. Parr , Phys. Rev. B 1988, 37, 785. 10.1103/physrevb.37.785 9944570

[82] A. D. Becke , Phys. Rev. A 1988, 38, 3098. 10.1103/physreva.38.3098 9900728

[83] R. Krishnan , J. S. Binkley , R. Seeger , J. A. Pople , J. Chem. Phys. 1980, 72, 650.

[84] T. Clark , J. Chandrasekhar , G. W. Spitznagel , P. von Ragué Schleyer , J. Comput. Chem. 1983, 4, 294.

[85] J. Tomasi , B. Menucci , R. Cammi , Chem. Rev. 2005, 105, 2999. 16092826 10.1021/cr9904009

[86] Z. Wang , Chemistry (Basel, Switz.) 2024, 6, 1692.

[87] D. Geuenich , K. Hess , F. Köhler , R. Herges , Chem. Rev. 2005, 105, 3758. 16218566 10.1021/cr0300901

[88] W. J. Hehre , R. Ditchfield , J. A. Pople , J. Chem. Phys. 1972, 56, 2257.

[89] S. R. Forrest , D. D. C. Bradley , M. E. Thompson , Adv. Mater. 2003, 15, 1043.

[90] N. C. Greenham , R. H. Friend , D. D. C. Bradley , Adv. Mater. 1994, 6, 491.

[91] L. Bennett , B. Proppe , B. Melchers , 2020, 10.17169/refubium-26754.

