# Supplementary material for: Highly Emissive Hexa‐peri‐benzocoronene‐fluoranthene Hybrid as Easily Processable and Stable OLED Material
Source: Chemistry. 2025 May 3;31(30):e202500742. doi: 10.1002/chem.202500742 (PMC12117179; doi:10.1002/chem.202500742)
Supplement: Supplementary file 1 — Supporting Information [file CHEM-31-e202500742-s001.pdf]

Supporting Information

Table of Contents

1 General Information 2

2 Synthetic Procedures 4

3 Crystallographic Data 7

4 UV/Vis and Fluorescence Measurements 10

5 Photoluminescence Quantum Yield and Lifetime Measurements 13

6 Cyclic Voltammetry 15

7 Ultraviolet Photoelectron Spectroscopy (UPS) 17

8 Computational Details 18

8.1 Electronically Excited States 18

8.2 Racemization Barrier 19

8.3 NICS Calculations 19

8.4 AICD Calculations 20

9 Organic Light-Emitting Diode (OLED) 21

9.1 Device Fabrication 21

9.2 Device Performance 21

10 References 25

11 NMR Spectra 27

12 Optimized Geometries 32

1 General Information

All reagents were purchased from commercial sources and were used without further purification unless otherwise stated. All air and moisture sensitive reactions were performed under standard Schlenk technique using nitrogen as inert gas. Dry ethanol, DCM, THF, and toluene were obtained from MBraun Solvent Purification System MB-SPS 800 filled with Al_2O_3. CS_2 was purchased from Fisher Scientific.

Thin-layer chromatography was performed on ALUGRAM® Xtra SIL G/UV_{254} plates from Macherey-Nagel. Indication was performed by irradiation with UV light of wavelengths λ = 254 and 365 nm. Column chromatography was performed with silica gel Silica 60 M (0.04-0.063 mm) from Macherey-Nagel.

NMR spectra were recorded on a Bruker AVANCE III 500 (^{1}H 500 MHz, ^{13}C 126 MHz), a JEOL ECP 500 (^{1}H 500 MHz, ^{13}C 151 MHz), a JEOL ECZ 600 (^{1}H 600 MHz, ^{13}C 151 MHz), and a Bruker AVANCE700 (^{1}H 700 MHz, ^{13}C 176 MHz) spectrometer at 25 °C. The chemical shifts δ are calibrated on the respective residual solvent signal as internal standard. All shifts are reported in ppm and NMR multiplicities are abbreviated as s (singlet), d (duplet), t (triplet), q (quartet), m (multiplet). Coupling constants J are reported in Hz. NMR spectra were processed using the software MestReNova (version 14.3.0) from Mestrelab Research.

UV/Vis spectra of solutions were recorded on a Lambda 365 UV/Vis Spectrometer from Perkin Elmer. **UV/Vis spectra of films** were recorded on a Lambda 950 UV/Vis Spectrometer from Perkin Elmer. Thin films were spin coated onto cleaned quartz glass substrates according to the device fabrication procedure. The absorption was calculated from the transmission. **Photoluminescence spectra of solutions** were recorded on a FL 6500 Fluorescence Spectrometer from Perkin Elmer. UV/Vis and photoluminescence spectra were measured in quartz glass cuvettes from Hellma GmbH with 1 cm path length. **Photoluminescence spectra of thin films** were obtained on a FLS980 spectrometer from Edinburgh Instruments. The excitation wavelength was set to 470 nm.

Samples for **UPS** measurements were prepared by spin coating according to the device fabrication procedure (Chapter 9.1) onto cleaned, ITO covered glass substrate. The samples were illuminated using a monochromatized helium discharging lamp (21.22 eV). The energy of the electrons was analyzed using a SPECS PHOIBOS 100 hemisperical analyzer. A bias of -10 V was applied to clear the analyzer work function and access the secondary electron cut-off spectra. During the measurement, the sample was illuminated with visible light to avoid charging of the sample.

IR spectra were recorded on a FT-IR Spectrum Two with $LiTaO_3$ detector from Perkin Elmer.

HR-ESI mass spectra were measured on a Bruker timsTOF Pro mass spectrometer using a nano-ESI source.[1]

Elemental analysis was performed on a VARIO EL from Elementar. Measurement and evaluation were carried out by the analytical department.

Photoluminescence quantum yields (Φ) were determined absolutely with an integrating sphere setup from Hamamatsu (Quantaurus-QY C11347-11). All Φ measurements were performed at 25 °C using special 10 mm x 10 mm long neck quartz cuvettes from Hamamatsu. The **fluorescence lifetime** (τ) was recorded on a fluorometer FLS 920 (Edinburgh Instruments) equipped with a Hamamatsu R3809U-50 (range 200–850 nm, response width <25 ps), Multi-Channel Plate (MCP) detector, Czerny-Turner double monochromators and an Edinburgh Instruments EPL-375 (picosecond pulsed light emitting diode) for excitation at 375

nm. All the measurements were performed at T = 298 K using 10 mm x 10 mm quartz cuvettes from Hellma GmbH. Before each measurement, the instrument response function (IRF) was measured. The lifetime measurements were analyzed with Edinburgh Instruments FAST Software and fitted with a reconvolution fit. All the lifetimes could be evaluated mono, bi- or tri-exponentially with a reduced X^2 between 1.41 and 1.47.

Cyclic voltammetry experiments were performed on an Interface 1010B potentiostat from Gamry Instruments using a three-electrode setup consisting of a platinum working electrode (3 mm diameter), a platinum wire counter electrode and an Ag/AgCl reference electrode (3M NaCl). All solutions were prepared in dry solvents and degassed with nitrogen before the measurement. Measurements were performed at room temperature at a scan rate of 150 mV/s with a sample concentration of 2 mmol/L and a supporting electrolyte (n-Bu_4NPF_6) concentration of 0.1 mol/L. All measurements were rescaled using the Fc/Fc^+ redox couple as internal reference.

Single crystal structure data were acquired using a Bruker APEX-II CCD diffractometer with a rotating anode (Mo *Kα* radiation, λ = 0.71 Å or Cu *Kα* radiation, λ = 1.54 Å). Structural refinement was performed with the help of the programs Olex^2 from OlexSys Ltd.[2] and PLATON[3] using the packages SHELXS and SHELXL.[4] All non-hydrogen atoms were refined anisotropically. All hydrogen atoms were included into the model at geometrically calculated positions and refined using a riding model.

2 Synthetic Procedures

The synthetic pathway towards the precursor **1** is depicted in Figure S1. Compounds **S2**-**S3**, and **S6** were commercially available. The synthetic protocols and spectroscopic data are in accordance with the literature.[5]

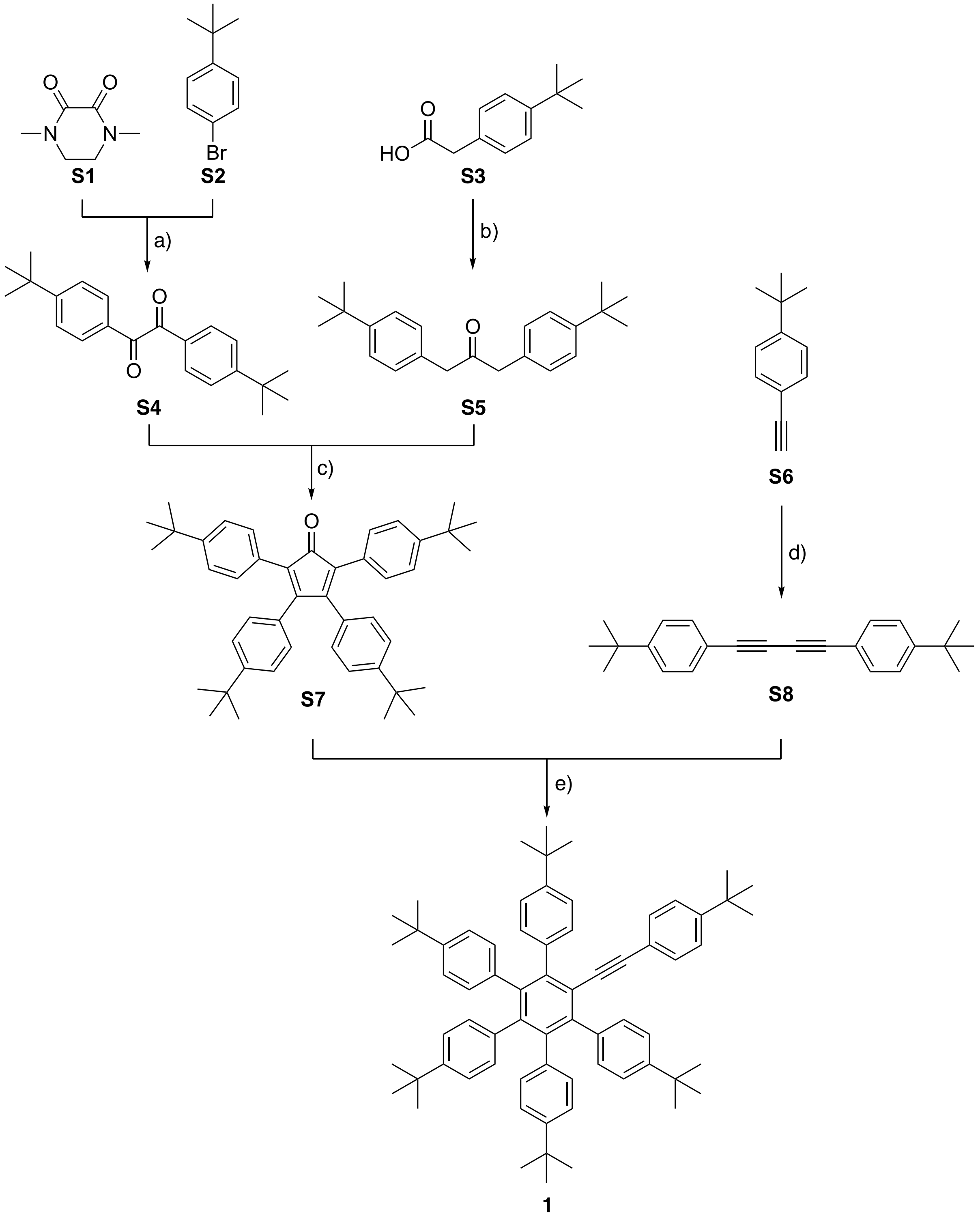

Figure S1: Synthetic pathway towards precursor **1**. Reaction conditions: a) *n*-BuLi, THF, 20 °C, 30 min, b) DCC, DMAP, DCM, rt, 24 h, c) KOH, EtOH, 78 °C, 15 min, d) CuI, $BnNH_2$, O_2, rt, 24 h, e) Ph_2O, N_2, 350 °C, 3 h.

2,5,8,11,17,19-Hexa-*tert*-butyldibenzo[*fg*,*ij*]indeno[1',2',3':8,9]pyreno[5,4,3,2,1-*pqrst*]-pentaphene (**3**)

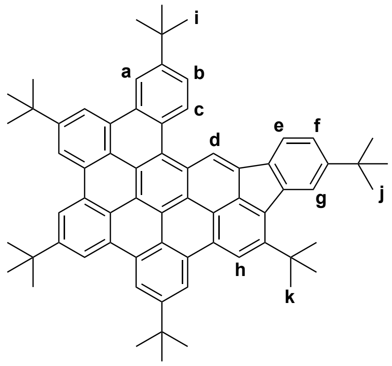

Protocol using $FeCl_3$ for oxidation:

Phenylbenzene **1** (500 mg, 0.56 mmol, 1 eq.) was dissolved in anhydrous degassed DCM (250 mL). At 0 °C, $FeCl_3$ (1.63 g, 10.0 mmol, 18 eq.) in freshly distilled anhydrous degassed $MeNO_2$ (25 mL) was added dropwise, and the solution thoroughly degassed with nitrogen. The mixture was stirred for 17 h at 0 °C. The reaction was then quenched by the addition of MeOH (100 mL). Water (100 mL) was added, the reaction mixture was washed with $NaHCO_3$ (2x 100 mL) and water (100 mL), dried over $MgSO_4$, filtered, and the solvent was removed under reduced pressure. Purification by column chromatography (CS_2, $R_f = 0.42$) yielded 371 mg compound **3** (0.42 mmol, 75%) as orange solid.

Protocol using Rathore's condition for oxidation:

Phenylbenzene **1** (200 mg, 0.22 mmol, 1 eq.) was dissolved in anhydrous degassed DCM (150 mL). In a second flask, 2,3-dichloro-5,6-dicyano-1,4-benzoquinone (DDQ) (608 mg, 2.68 mmol, 12 eq.) was dissolved in DCM (20 mL) and trifluoromethanesulfonic acid (TfOH) (1 mL). The solution of DDQ was added dropwise to the solution of **1** at -40 °C. The reaction mixture was stirred for 16 h at 0 °C and subsequently quenched by the addition of satd. aqueous $NaHCO_3$ (100 mL). The phases were separated, and the aqueous mixture was extracted with DCM (3x 50 mL). The combined organic phases were washed with water (100 mL), dried over $MgSO_4$, filtered, and the solvent was removed under reduced pressure. Purification by column chromatography (CS_2, $R_f = 0.42$) yielded 172 mg compound **3** (0.19 mmol, 87%) as orange solid.

^{1}H NMR (500 MHz, $CDCl_3$, rt, ppm): δ = 9.93 (s, 1H, **d**), 9.32 (s, 1H, **h**), 9.32–9.30 (m, 2H), 9.29 (d, J = 1.7 Hz, 1H), 9.18 (d, J = 1.1 Hz, 1H), 9.17 (d, J = 1.4 Hz, 1H), 9.12 (d, J = 8.5 Hz, 1H, **c**), 9.12 (d, J = 1.5 Hz, 1H), 8.99 (d, J = 2.0 Hz, 1H, **a**), 8.61 (d, J = 1.6 Hz, 1H, **g**), 8.34 (d, J = 7.8 Hz, 1H, **e**), 7.92 (dd, J = 8.5, 2.0 Hz, 1H, **b**), 7.59 (dd, J = 7.8, 1.6 Hz, 1H, **f**), 2.07 (s, 9H, **k**), 1.84 (s, 9H, ***t*-Bu**), 1.82 (s, 9H, ***t*-Bu**), 1.80 (s, 9H, ***t*-Bu**), 1.70 (s, 9H, **i**), 1.57 (s, 9H, **j**).

^{13}C NMR (151 MHz, $CDCl_3$, rt, ppm): δ = 150.8 (**C_q**), 150.1 (**C_q**), 149.5 (**C_q**), 149.4 (**C_q**), 147.7 (**C_q**), 140.9 (**C_q**), 137.7 (**C_q**), 134.4 (**C_q**), 132.9 (**C_q**), 132.1 (**C_q**), 131.9 (**C-H**), 131.8 (**C_q**), 131.0 (**C_q**), 130.9 (**C_q**), 130.8 (**C_q**), 130.3 (**C_q**), 130.3 (**C_q**), 130.2 (**C_q**), 130.2 (**C_q**), 128.6 (**C_q**), 126.3 (**C_q**), 125.5 (**C-H**), 125.2 (**C_q**), 124.5 (**C-H**), 124.0 (**C_q**), 123.8 (**C_q**), 123.8 (**C_q**), 123.2 (**C-H**), 122.4 (**C_q**), 122.0 (**C_q**), 121.6 (**C_q**), 121.5 (**C-H**), 121.4 (**C_q**), 121.0 (**C-H**), 120.9 (**C_q**), 120.1 (**C-H**), 119.6 (**C-H**), 119.4 (**C-H**), 119.4 (**C-H**), 119.3 (**C-H**), 119.2 (**C-H**), 119.0 (**C-H**), 118.5 (**C-H**), 36.6 (**C_q-k**), 35.9 (**C_q**), 35.9 (**C_q**), 35.8 (**C_q**), 35.6 (**C_q-j**), 35.4 (**C_q-i**), 32.1 (***t*-Bu**), 32.1 (***t*-Bu**), 32.1 (***t*-Bu**), 31.9 (**j**), 31.8 (**i**), 31.3 (**k**).

FT-IR (ATR, cm^{-1}): $\tilde{\nu}$ = 3071 (vw), 3008 (vw), 2953 (vs), 2903 (w), 2866 (w), 1761 (vw), 1748 (vw), 1606 (m), 1568 (w), 1477 (m), 1463 (m), 1448 (w), 1393 (m), 1362 (vs), 1309 (w), 1290 (w), 1256 (vs), 1246 (m), 1202 (w), 1109 (w), 1022 (w), 937 (w), 911 (w), 866 (vs), 845 (w), 828 (s), 811 (s), 752 (m), 660 (m), 630 (s), 621 (m).

HRMS (ESI): m/z = calc. 884.5321, meas. 884.5328 $[M]^{\cdot+}$.

EA: $C_{68}H_{68}$; calc. C, 92.26; H, 7.74; meas. C, 92.30; H, 7.808.

3 Crystallographic Data

The supplementary crystallographic data for compounds **1** and **3** can be found in the CCDC with the following deposit numbers CCDC 2401868 (**1**) and 2401878 (**3**). These data can be obtained free of charge from www.ccdc.cam.ac.uk/data_request/cif.

	**1**	**3**
Sample Name	AK161	AK164
CCDC number	2401868	2401878
Empirical formula	$C_{68}H_{78}$ + 3 (CH_2Cl_2)	$C_{68}H_{68}$
Formula Weight	1150.17	885.22
Temperature/K	120	140
Crystal system	triclinic	monoclinic
Space group	*P*-1	$P2_1/n$
a/Å	12.1864(4)	22.4496(2)
b/Å	16.2621(5)	16.6620(1)
c/Å	18.1151(6)	30.2958(2)
α/°	100.670(1)	90
β/°	106.692(1)	98.9653(3)
γ/°	101.698(1)	90
Volume/Å^3	3251.95(18)	11193.85(14)
Z	2	8
Reflections collected	64257	147619
Independent reflections (R_{int})	12324 (0.0460)	19714 (0.0495)
R_1 [I>2σ (I)]	0.0661	0.0693
wR_2 (all data)	0.1633	0.2038

Additional refinement details for compound 1

Crystals suitable for X-ray crystallographic analysis were grown by slow evaporation of a saturated solution of **1** in DCM. The compound crystallized together with three molecules dichloromethane per ASU making the crystal very labile and prohibiting longer measurements. The refinement was done with the Olex^2-solve method using spherical form factors. The slightly increased values for R_1 and wR_2 originate from the disorder of three *tert*-butyl groups being modeled with SADI and RIGU restraints as well as residual electron density in proximity to the solvent molecules. Hydrogen atoms in the disordered *tert*-groups were placed using AFIX 33.

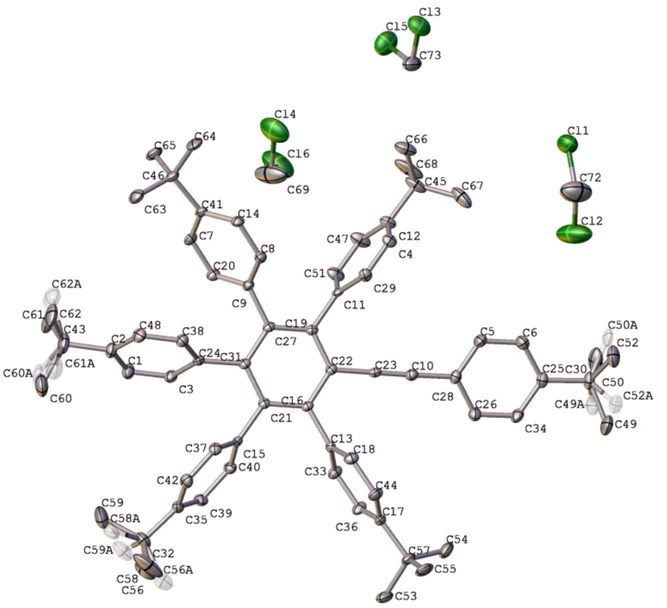

Figure S2: Structure of compound **1** in the solid state crystallized with three molecules dichloromethane. Hydrogen atoms omitted for clarity, disorder displayed transparent. Thermal ellipsoids shown at the 50% level.

Additional refinement details for 3

Crystals suitable for X-ray crystallographic analysis were grown by vapor diffusion of MeOH into a saturated solution of compound **3** in toluene. The increased values for R_1 and wR_2 originate from the disorder of five *tert*-butyl groups being modeled with SADI, SIMU and RIGU restraints. One disordered molecule toluene cocrystallized per ASU was modeled using SADI, SIMU and RIGU restraints. Two disordered molecules of MeOH were treated by using the Olex[2] solvent mask.

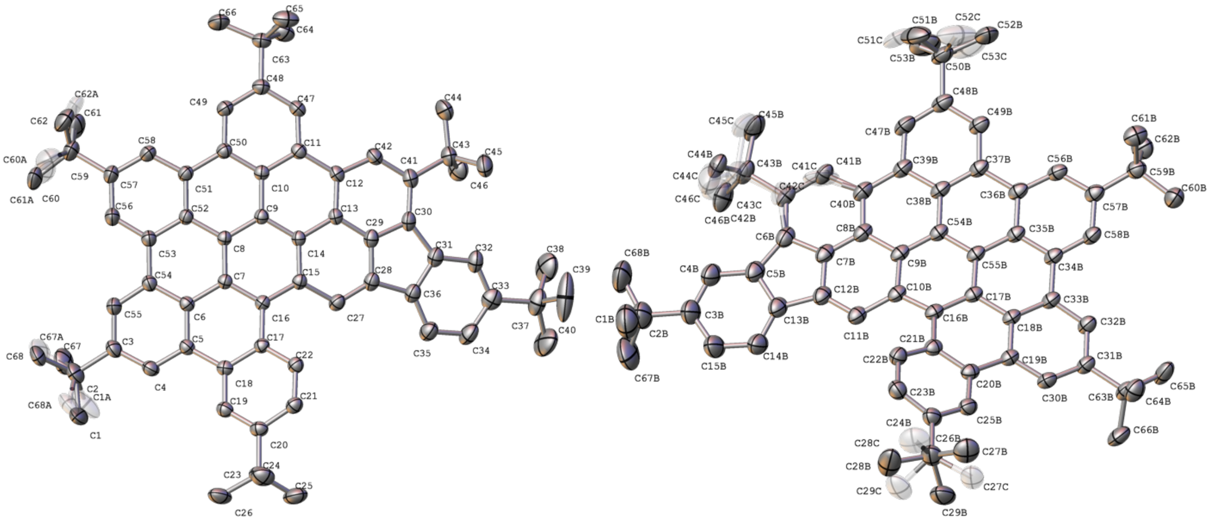

Figure S3: Structure of (*M*)-enantiomer (left) and (*P*)-enantiomer (right) of compound **3** in the solid state. Hydrogen atoms and solvent molecules omitted for clarity, disorder displayed transparent. Thermal ellipsoids shown at the 50% level.

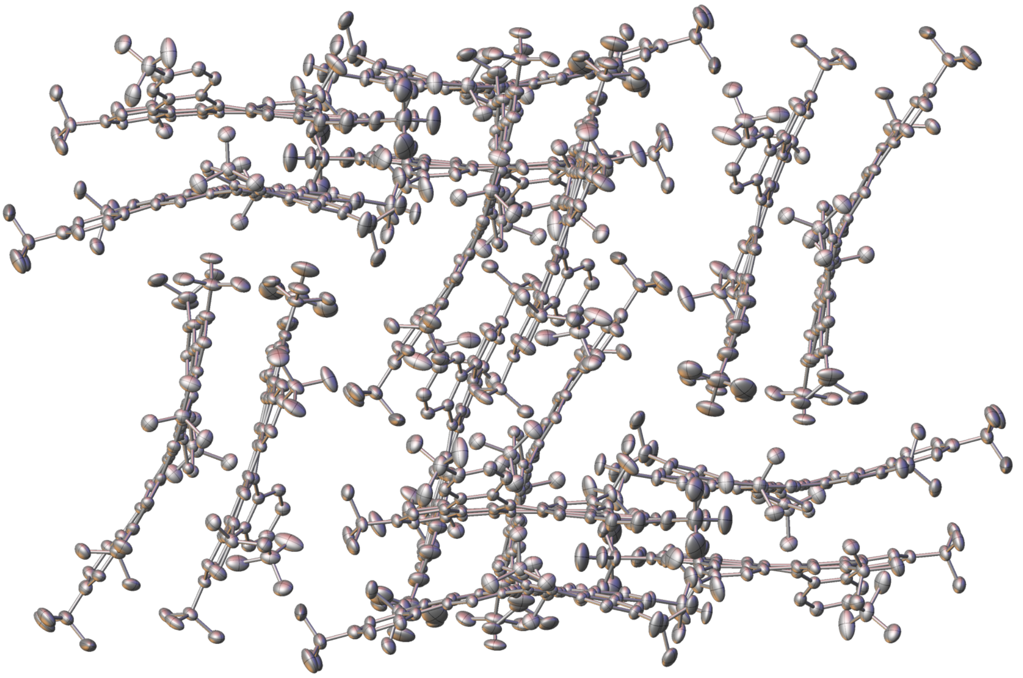

Figure S4: Packing of compound **3** in the solid state. Hydrogen atoms and solvent molecules omitted for clarity, major disorder part shown. Thermal ellipsoids shown at the 50% level.

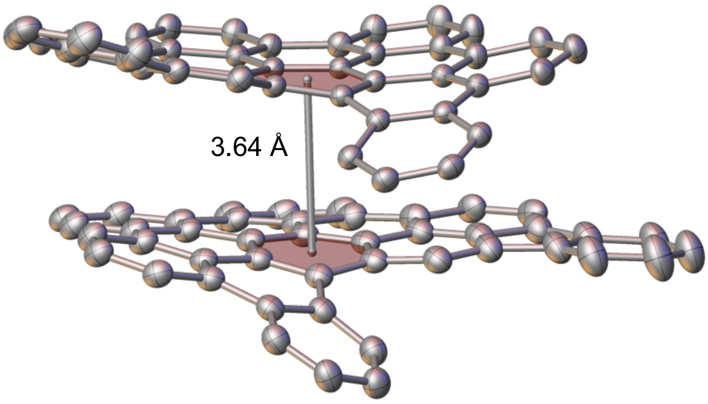

Figure S5: Asymmetric unit of the structure of compound **3** in the solid state with (*P*)- and (*M*)-enantiomer, and interplanar distance between the central rings. Hydrogen atoms, solvent molecules, *tert*-butyl groups and disorder omitted for clarity.

4 UV/Vis and Fluorescence Measurements

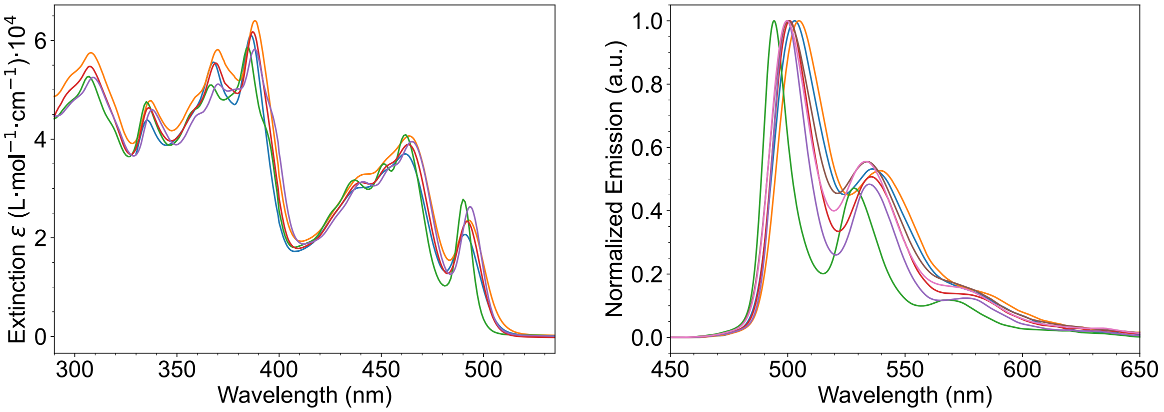

Figure S6: Absorption spectra (left) and normalized photoluminescence spectra (right) of compound **3** in acetone (blue), DCM (orange), *n*-Hexane (green), THF (red), toluene (purple), MeOH (brown) and EtOH (pink). Absorption spectra of MeOH and EtOH are plotted separately in Figure S7. The corresponding excitation and emission wavelengths and Stokes shifts are listed in Table S1.

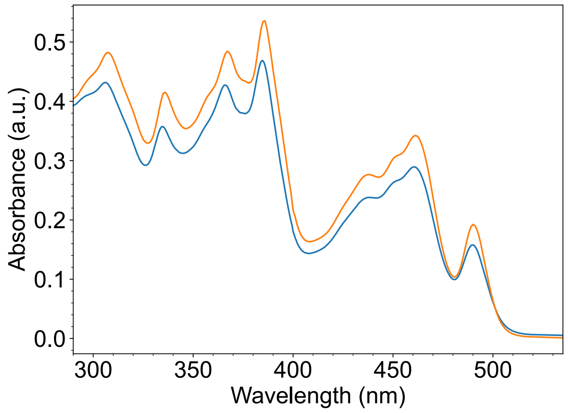

Figure S7: Absorption spectra of compound **3** in MeOH (blue) and EtOH (orange).

Table S1: Absorption and emission maxima with Stokes shift calculated from respective absorption and emission maxima marked with asterisk.

E_T^N	Solvent	Absorption Maxima (nm)	Emission Maxima (nm)	Stokes Shift (nm)
0.762	MeOH	227<br>306<br>335<br>366<br>385<br>438<br>451<br>461*<br>490	500*<br>534<br>578	39
0.654	EtOH	227<br>308<br>336<br>367<br>386<br>438<br>451<br>462*<br>490	500*<br>533<br>574	38

0.355	Acetone	335<br>368<br>387<br>439<br>451<br>462*<br>491	503*<br>536<br>580	41
0.309	DCM	237<br>308<br>337<br>370<br>388<br>442<br>464*<br>493	505*<br>540<br>582	41
0.207	THF	246<br>308<br>336<br>369<br>388<br>440<br>463*<br>492	501*<br>536<br>576	38
0.099	Toluene	309<br>338<br>370<br>388<br>441<br>455<br>465*<br>494	500*<br>535<br>576	35
0.009	*n*-Hexane	228<br>307<br>335<br>367<br>385<br>437<br>452<br>462*<br>490	494*<br>528<br>568	32

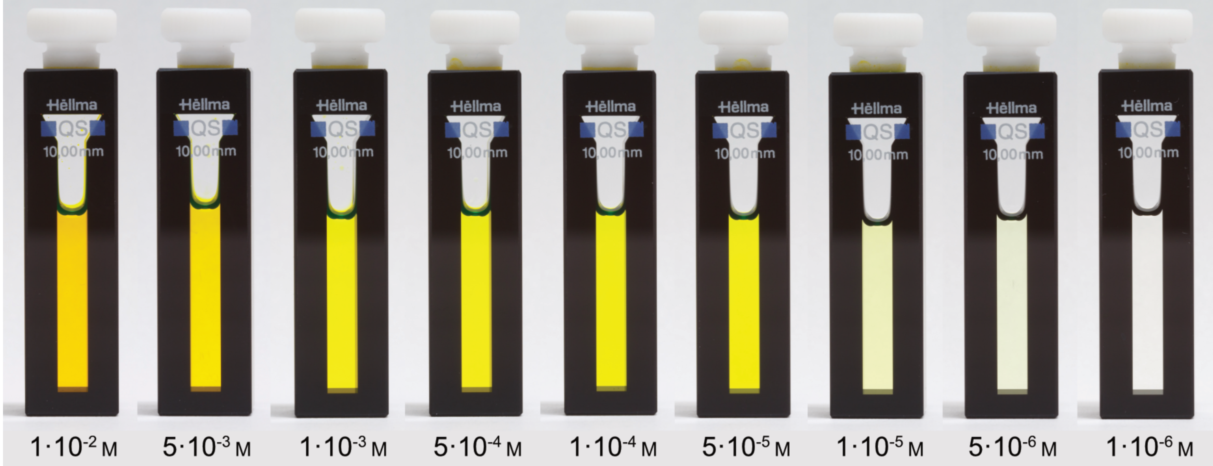

Figure S8: Color change of solutions of compound **3** in chloroform in dependence of the concentration in the range from c = $1 \cdot 10^{-2}$ to $1 \cdot 10^{-6}$ mol/L. The formation of aggregates can be observed by the overall more yellowish color (> 10^{-3} M) in contrast to the more green color of the monomeric species.

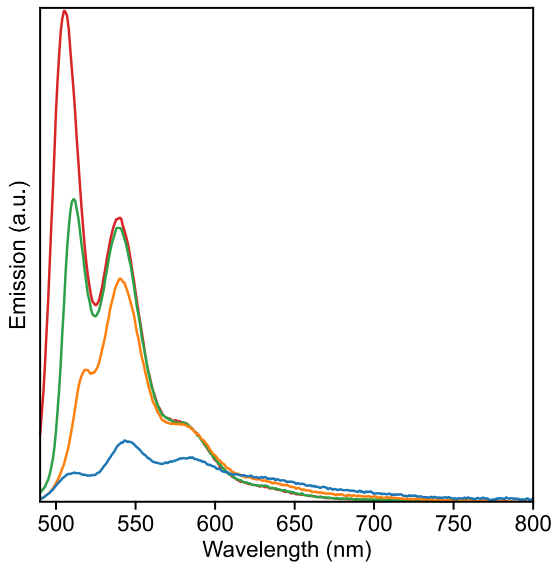

Figure S9: Concentration dependent photoluminescence spectra of compound **3** in chloroform solution, c = 10 mg/mL (blue), 1 mg/mL (orange), 0.1 mg/mL (green), 0.01 mg/mL (red). Self-absorption at higher concentrations significantly shifts the emission spectrum to higher wavelengths.

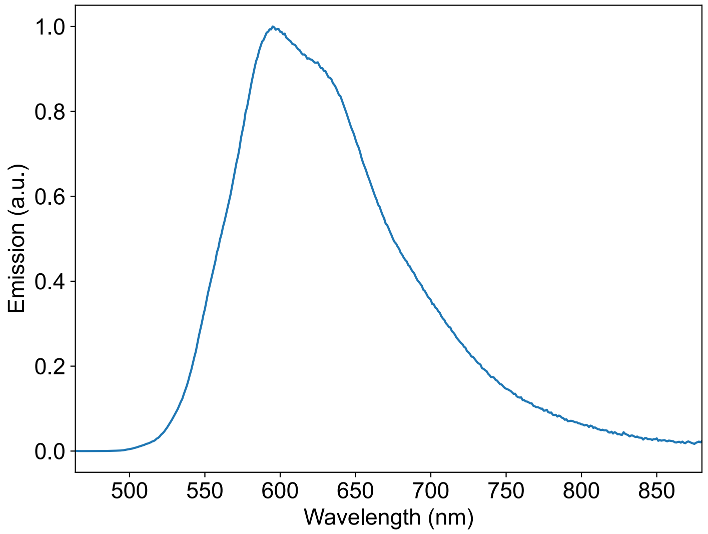

Figure S10: Emission spectrum of compound **3** in the solid state.

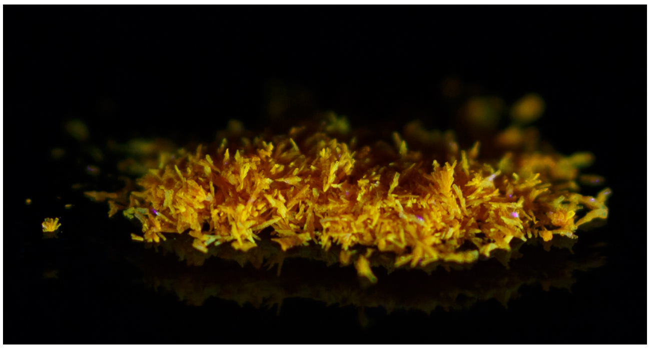

Figure S11: Picture of crystalline compound **3** in solid state.

5 Photoluminescence Quantum Yield and Lifetime Measurements

The measured photoluminescence quantum yields (Φ) and lifetimes (τ) are shown in Table S2.

Table S2: Photoluminescence quantum yields (Φ) and luminescence lifetimes (τ) in solvents of different polarity and solid, classified by the Dimroth-Reichardt parameter E_T^N.[6]

		***t*-Bu-HBC**[5f]		**2**[5f]		**3**	
E_T^N	**Solvent**	Φ [%]	τ [ns]	Φ [%]	τ [ns]	Φ [%]	τ [ns]
0.762	MeOH					57	8.2
0.654	EtOH	2	11.0	11	17.8	61	9.2
0.355	Acetone	–	–	–	–	61	7.6
0.309	DCM	3	14.2	16	21.4	51	5.6
0.207	THF	–	–	–	–	62	7.8
0.099	Toluene	–	–	–	–	67	7.3
0.009	*n*-Hexane	2	12.4	11	17.2	61	9.3
	Solid	2	–	–	–	4	4.8

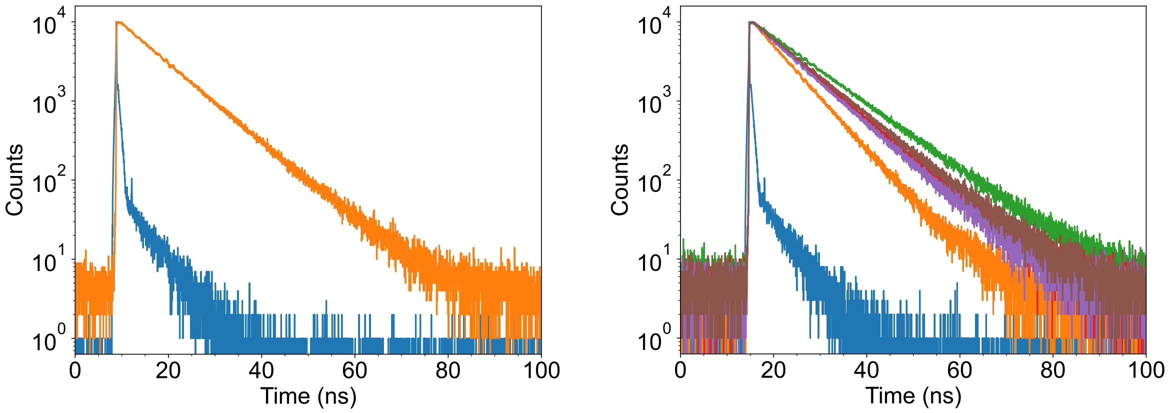

Figure S12: Luminescence lifetimes of compound **3** in acetone (orange, left), DCM (orange, right), *n*-hexane (green), THF (red), toluene (purple), and MeOH (brown), instrument response function (IRF) in blue. All measurements performed in solutions saturated with argon. Compound **3** was excited at 375 nm, the emission was measured at the maximum emission of the first fluorescence peak, respectively (s. Table S1).

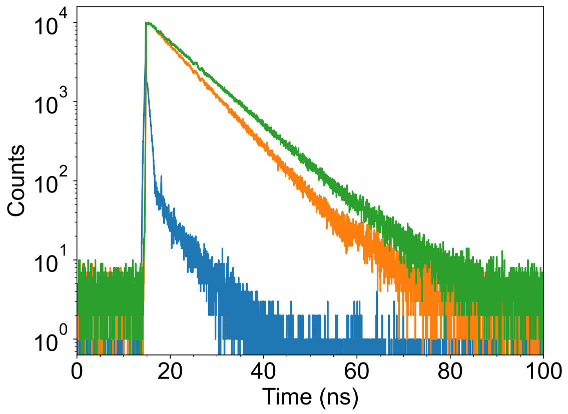

Figure S13: Luminescence lifetimes of compound **3** in EtOH (orange), instrument response function (IRF) in blue. All measurements performed in solutions saturated with argon. Compound **3** was excited at 375 nm, the emission was measured at the maximum emission of the first fluorescence peak, respectively (s. Table S1).

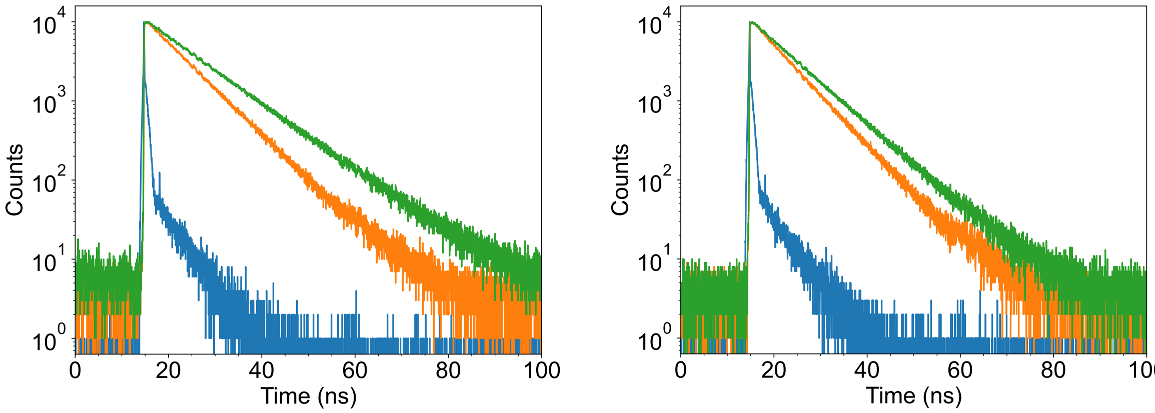

Figure S14: Luminescence lifetimes of compound **3** in *n*-hexane (left) and toluene (right). Argon-saturated solutions in green, equilibrated with air in orange, instrument response function (IRF) in blue. Compound **3** was excited at 375 nm, the emission was measured at the maximum emission of the first fluorescence peak, respectively (s. Table S1).

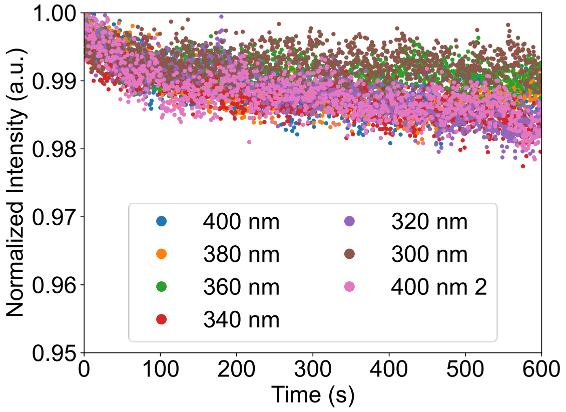

Figure S15: Photostability measurements of compound **3** in toluene equilibrated with air at a concentration of $1.0 \cdot 10^{-6}$ mol/L under irradiation with different wavelengths using a 450 W Xe discharge lamp. All measurements were performed at a temperature of 22 °C, solutions were stirred during irradiation.

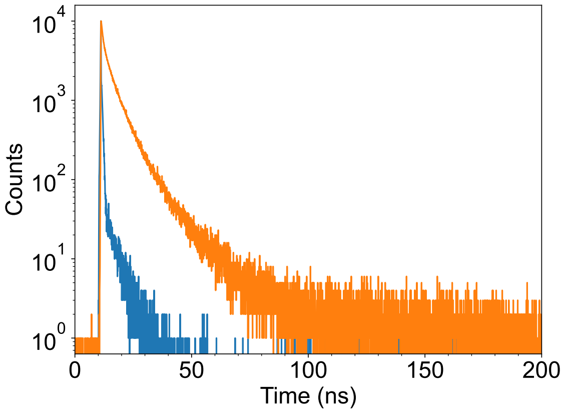

Figure S16: Luminescence lifetime (τ) of compound **3** in the solid state (orange). Instrument response function (IRF) in blue. The measurement was performed at room temperature under air. Compound **3** was excited at 375 nm, the emission was measured at the maximum emission of the first fluorescence peak, 596 nm (s. Figure S10).

6 Cyclic Voltammetry

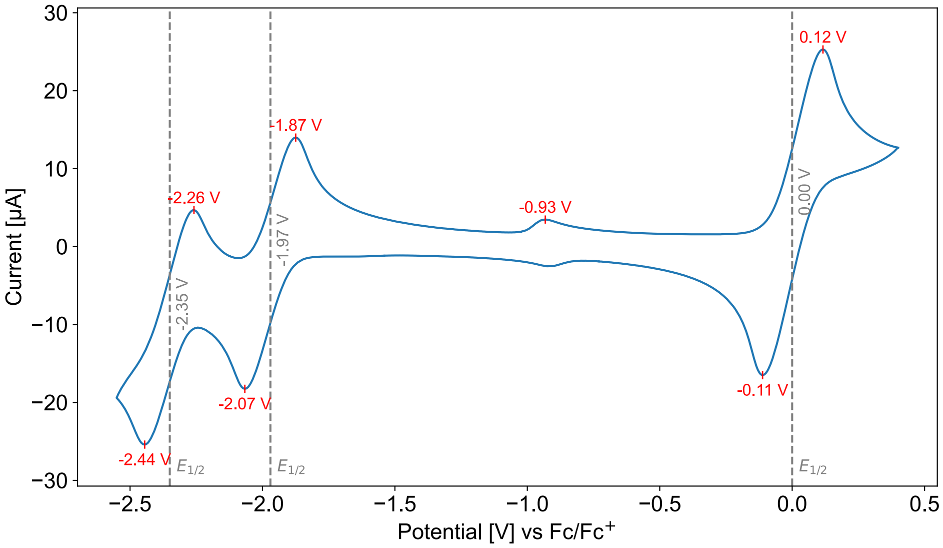

Figure S17: Cyclic voltammogram of the reductions of compound **3** (c = 2 mmol/L) measured in THF with 0.1M *n*-Bu_4NPF_6 as supporting electrolyte. The voltammogram is referenced against the Fc/Fc^+ redox couple ($E_{1/2}$ = 0.00 V).

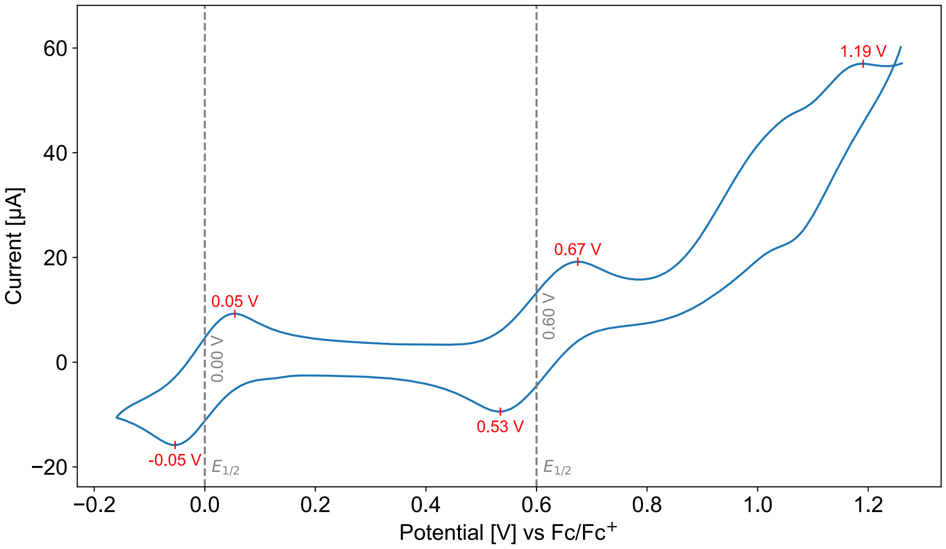

Figure S18: Cyclic voltammogram of the oxidations of compound **3** (c = 2 mmol/L) measured in DCM with 0.1M *n*-Bu_4NPF_6 as supporting electrolyte. The voltammogram is referenced against the Fc/Fc^+ redox couple ($E_{1/2}$ = 0.00 V).

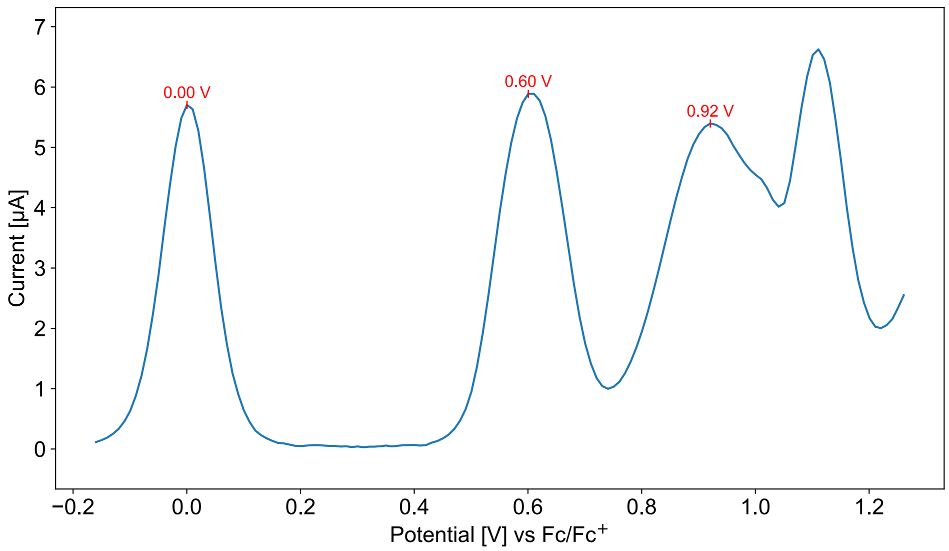

Figure S19: Differential pulse voltammogram (DPV) of compound **3** in DCM. The voltammogram is referenced against the Fc/Fc^{+} redox couple ($E_{1/2}$ = 0.00 V).

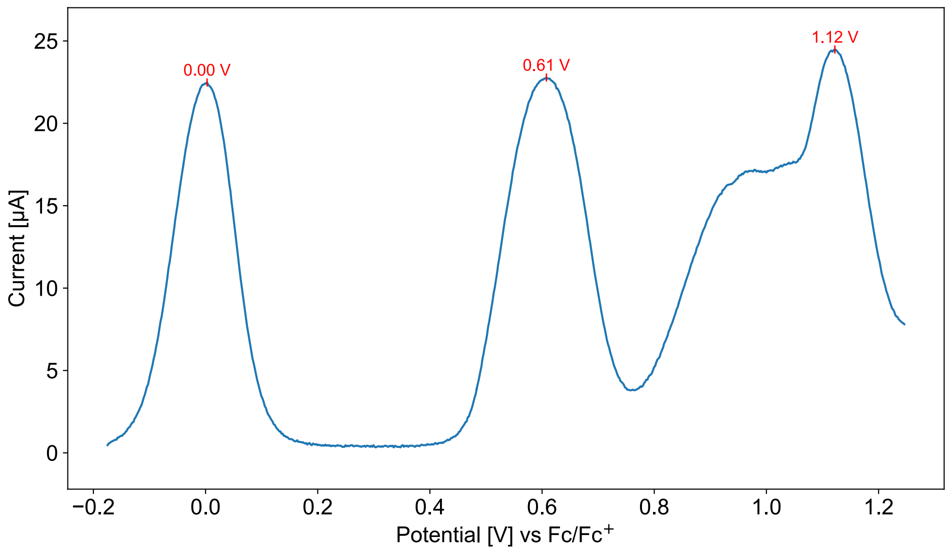

Figure S20: Squarewave voltammogram (SWV) of compound **3** in DCM. The voltammogram is referenced against the Fc/Fc^{+} redox couple ($E_{1/2}$ = 0.00 V).

7 Ultraviolet Photoelectron Spectroscopy (UPS)

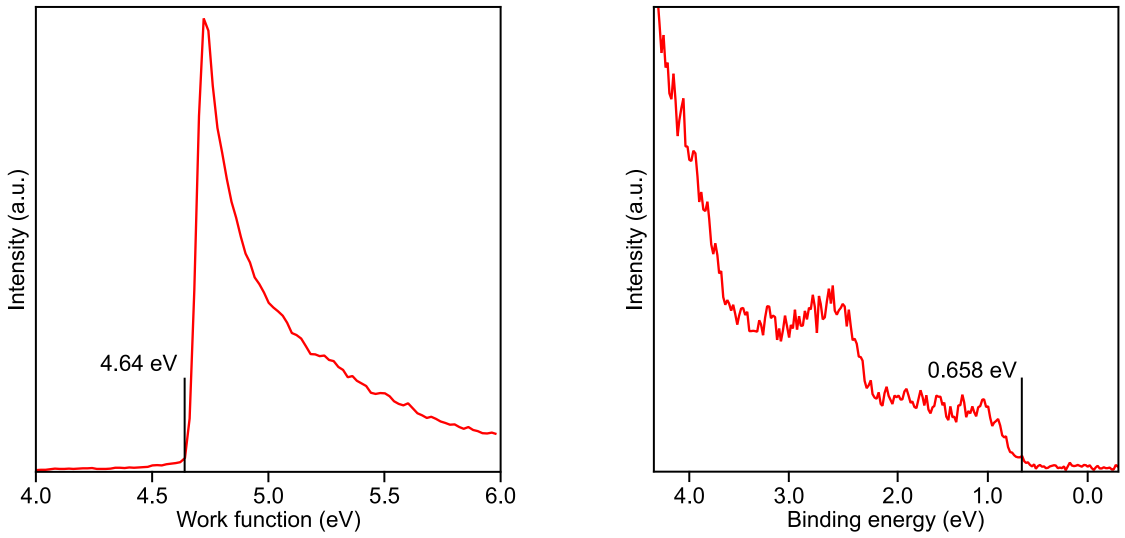

Figure S21: UPS spectra of a spin coated film of compound **3**. Left: SECO region, right: valence region. Work function 4.64 eV, HOMO positioned at 0.66 eV.

8 Computational Details

Quantum chemical calculations were performed using the ORCA 6.0.0 program package and the Gaussian16 software to investigate the absorption and emission properties of the compound as well as the electron delocalization and aromatic behavior of the synthesized compounds.[7]

Structure optimizations were performed with ORCA at the ωB97X-D3BJ/def2-TZVP level of density functional theory (DFT) using default convergence criteria.[8] The optimized structures were validated with frequency analysis.

With these optimized structures, time-dependent DFT (TD-DFT) calculations were performed at the B3LYP/6-311+G** level of theory with the Grimme D3 dispersion correction using the Gaussian16 software.[7b,9] The functional was selected after intensive testing to align reasonably with the experimental absorption spectra. Solvent effects were considered by using the Polarizable Continuum Model (PCM) with dichloromethane (ε = 8.93) as solvent.[10]

Nucleus independent chemical shift (NICS) and anisotropy of the induced current density (AICD) were calculated at the B3LYP/def2-TZVP level of theory using the Gaussian16 software of theory starting from the optimized ground state geometries. NICS input files were generated using the py.Aroma 4 software package,[11] $NICS_{ZZ}$ corrections for tilted rings were computed with the same software. AICD plots were generated using the software package from Herges *et al.*[12] The respective *π*-orbitals used for AICD plots are listed in section 8.4.

8.1 Electronically Excited States

Table S3: Electronically excited singlet states of compound **3** with energy, oscillator strength and major orbital contributions (>10%) calculated using time-dependent DFT (TD-DFT) at the B3LYP/6-311+G** level of theory in Gaussian16 using the structures optimized with ORCA at the ωB97X-D3BJ/def2-TZVP level of theory.

Excited State	Energy (eV)	Wavelength (nm)	Oscillator Strength f_{osc}	Major Orbital Contributions
1	2.6749	463.51	0.5328	89% HOMO-LUMO
2	2.7989	442.97	0.2334	76% HOMO(-1)-LUMO<br>12% HOMO-LUMO(+1)
3	3.0473	406.87	0.0148	92% HOMO(-2)-LUMO
4	3.1772	390.23	0.5318	84% HOMO-LUMO(+1)<br>13% HOMO(-1)-LUMO
5	3.3255	372.83	0.0276	40% HOMO(-3)-LUMO<br>27% HOMO-LUMO(+2)<br>23% HOMO(-1)-LUMO(+1)
6	3.3661	368.33	0.4262	62% HOMO(-1)-LUMO(+1)<br>31% HOMO(-2)-LUMO
7	3.5475	349.49	0.1098	44% HOMO-LUMO(+2)<br>19% HOMO(-3)-LUMO<br>12% HOMO(-2)-LUMO(+1)
8	3.6104	343.41	0.1358	47% HOMO(-1) -LUMO(+2)<br>18% HOMO-LUMO(+2)<br>17% HOMO(-2) -LUMO(+1)
9	3.7562	330.08	0.4287	52% HOMO(-2)-LUMO(+1)<br>30% HOMO(-1)-LUMO(+2)
10	3.7917	326.99	0.0489	70% HOMO-LUMO(+3)<br>10% HOMO(-5)-LUMO
11	3.8597	321.23	0.0024	56% HOMO(-4)-LUMO<br>13% HOMO-LUMO(+3)

12	3.8910	318.65	0.0348	36% HOMO(-3)-LUMO(+1)<br>23% HOMO(-5)-LUMO<br>18% HOMO(-4)-LUMO
13	3.9357	315.03	0.1209	40% HOMO(-3)-LUMO(+1)<br>39% HOMO(-5)-LUMO
14	3.974	311.99	0.0061	48% HOMO(-6)-LUMO<br>19% HOMO-LUMO(+5)<br>14% HOMO(-4)-LUMO
15	4.0053	309.55	0.0143	53% HOMO(-1)-LUMO(+3)<br>21% HOMO-LUMO(+5)<br>14% HOMO-LUMO(+4)

8.2 Racemization Barrier

All calculations were performed on the B3LYP/6-31G(d)[13] level of theory using the Gaussian16 program package. Here, a smaller basis set was used to reduce the run time of the calculations. To investigate the racemization barrier of compound **3**, we performed a relaxed redundant coordinate scan by varying the dihedral angle of the [4]helicene unit (C16-C15-C34-C44) starting from the calculated ground state geometry. The discontinuities in the curve are caused by a flip of the aromatic backbone resulting in a strain release and energy gain. The applicability of this method for small helicene systems was shown before.[5f]

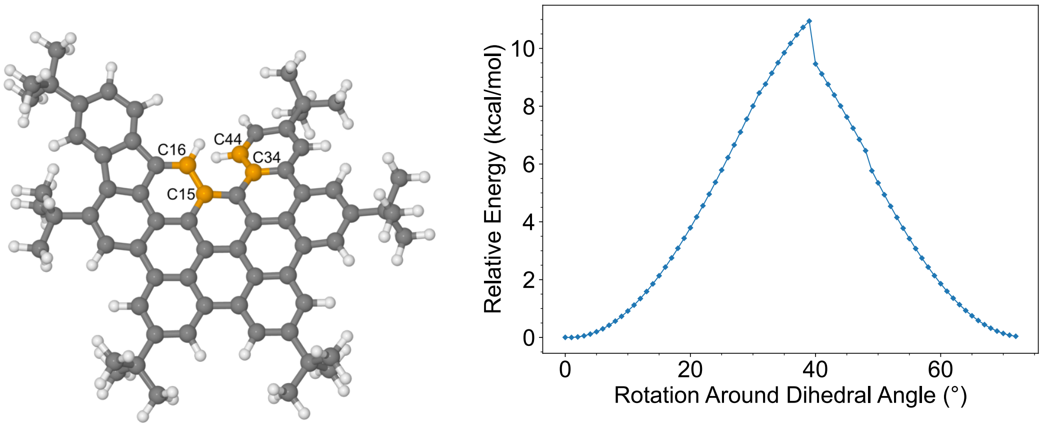

Figure S22: Left: Calculated ground state geometry with atoms used in the redundant coordinate scan highlighted in orange. Right: Racemization energy of compound **3** calculated at the B3LYP/6-31G(d) level of theory in dependence of the dihedral angle of the [4]helicene unit.

8.3 NICS Calculations

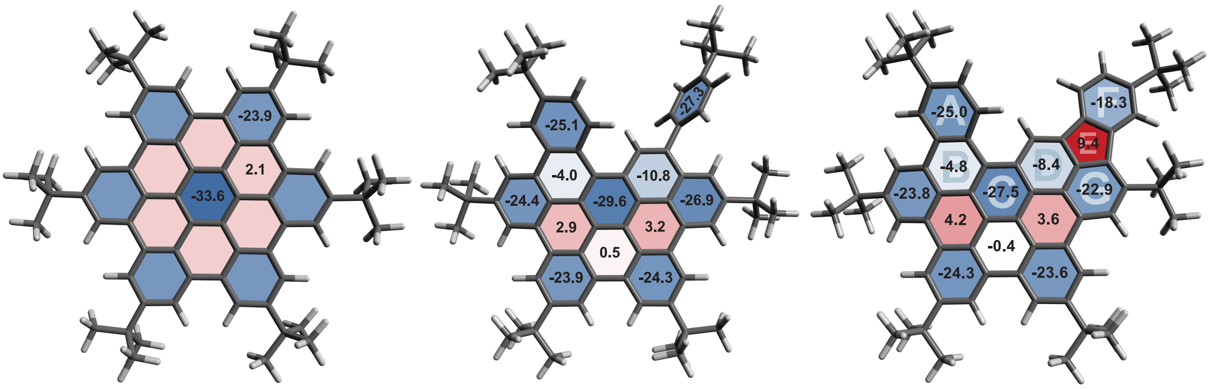

Figure S23: NICS(1)$_{ZZ}$ values of *t*-Bu-HBC (left) and compound **2** (middle) and compound **3** (right) calculated at the B3LYP/def2-TZVP level of theory.

8.4 AICD Calculations

Table S4: Numbers of orbitals with main contribution to the π-system used for the generation of AICD plots.

	***t*-Bu-HBC**	**2**	**3**
**Orbital Numbers**	231<br>230<br>229<br>228<br>227<br>226<br>224<br>223<br>222<br>221<br>218<br>217<br>216<br>210<br>204<br>196<br>194<br>190<br>171<br>166<br>165<br>164	239<br>238<br>237<br>236<br>235<br>234<br>233<br>232<br>231<br>230<br>229<br>228<br>227<br>226<br>223<br>203<br>201<br>198<br>197	238<br>237<br>236<br>235<br>234<br>233<br>232<br>231<br>230<br>229<br>228<br>227<br>226<br>225<br>201<br>197<br>168

9 Organic Light-Emitting Diode (OLED)

9.1 Device Fabrication

For the OLED fabrication, pre-patterned indium tin oxide (ITO) substrates (Psiotec Ltd.) were first cleaned by sonication in acetone and then isopropanol. To further clean and activate the surface, they were treated with an oxygen plasma for 5 min.

The hole injection material PEDOT:PSS (AI 4083, Ossila) was then spin coated on the substrates (1000 rpm, 12 s, 2000 rpm, 30 s) and baked at 220 °C for 15 min to form a 50-60 nm thick layer.

Poly(9,9-dioctylfluorene-alt-*N*-(4-*sec*-butylphenyl)-diphenylamine) (TFB) was spin coated as a hole transport and electron blocking layer. After spin coating from a 3 mg/mL toluene solution at 6000 rpm for 45 s, the film was placed in a vacuum oven at 200 °C for 1 h.

For the emitter layer, different chloroform solutions containing either just the emitter molecule at 5 mg/mL or a mixture of the emitter and host molecules in different ratios were spin coated. Smooth, closed films were achieved when spin coating at 1500 rpm for 30 s. Spin coating was performed in ambient conditions.

1,3,5-Tri(*m*-pyridin-3-yl-phenyl)benzene (TmPyPB) was used as an electron transport and hole blocking layer. It was deposited by thermal deposition in a physical vapor deposition chamber (Angstrom Engineering Inc.) at a base pressure below $1 \cdot 10^{-7}$ mbar. The top electrode was fabricated *via* thermal evaporation, consisting of a 5 nm thick calcium (Ca) layer and a 200 nm thick aluminum (Al) layer. A shadow mask was employed to create eight pixels with an active area of 0.04 cm^2 each on one substrate.

The completed devices were encapsulated with UV-curable resin (Ossila) and glass cover slips.

9.2 Device Performance

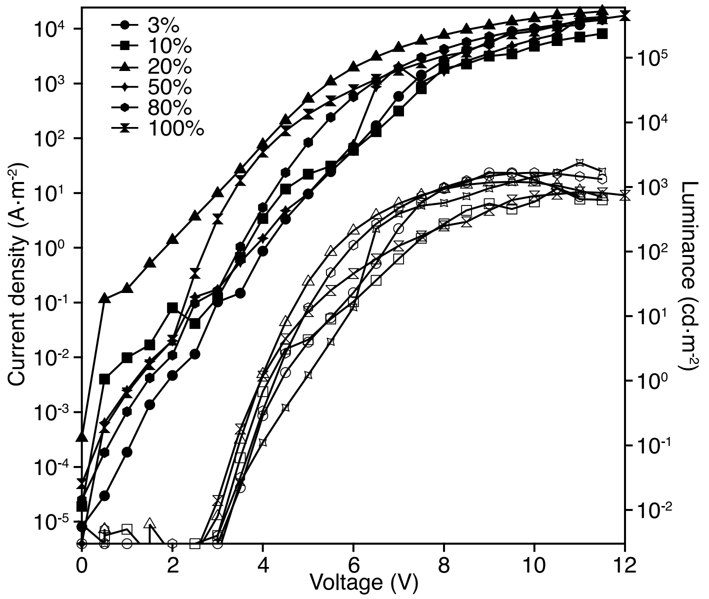

Figure S24: JVL characteristics of OLED with varying amounts of emitter material in the host. The turn-on behavior and luminance values are in a similar range for all devices. Current density shown with filled markers, luminance without fill.

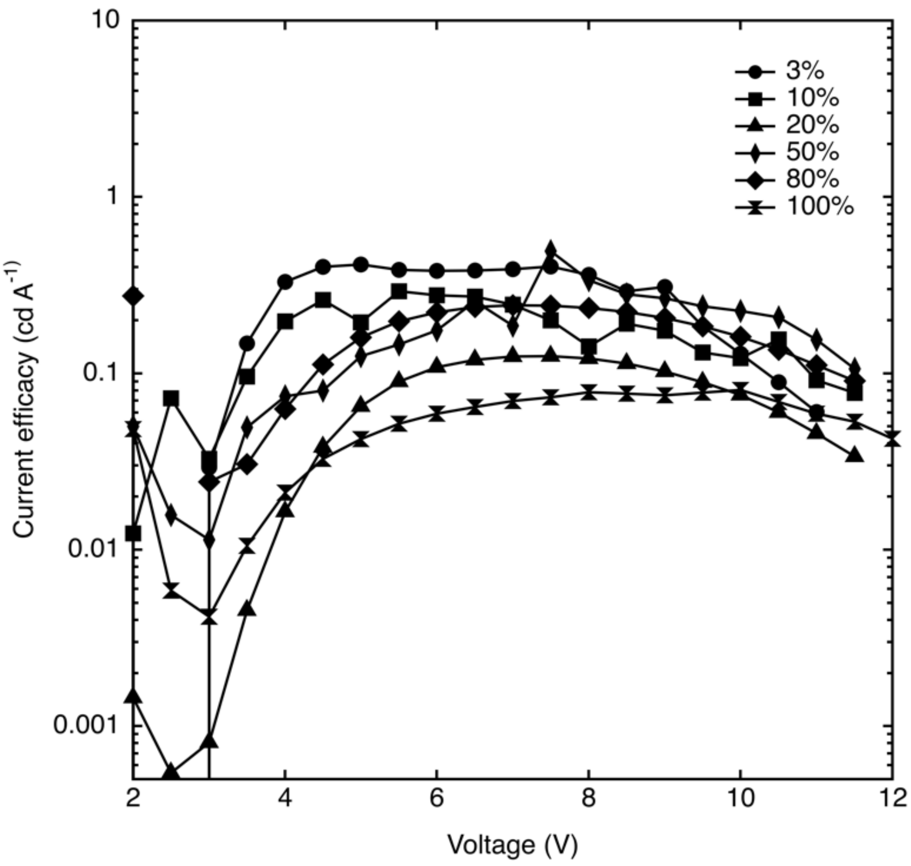

Figure S25: Current efficacies for the OLEDs with varying amounts of emitter in the host.

The current efficacies increase after the turn-on and then plateau. The devices show only minor roll-off at voltages higher than 10 V.

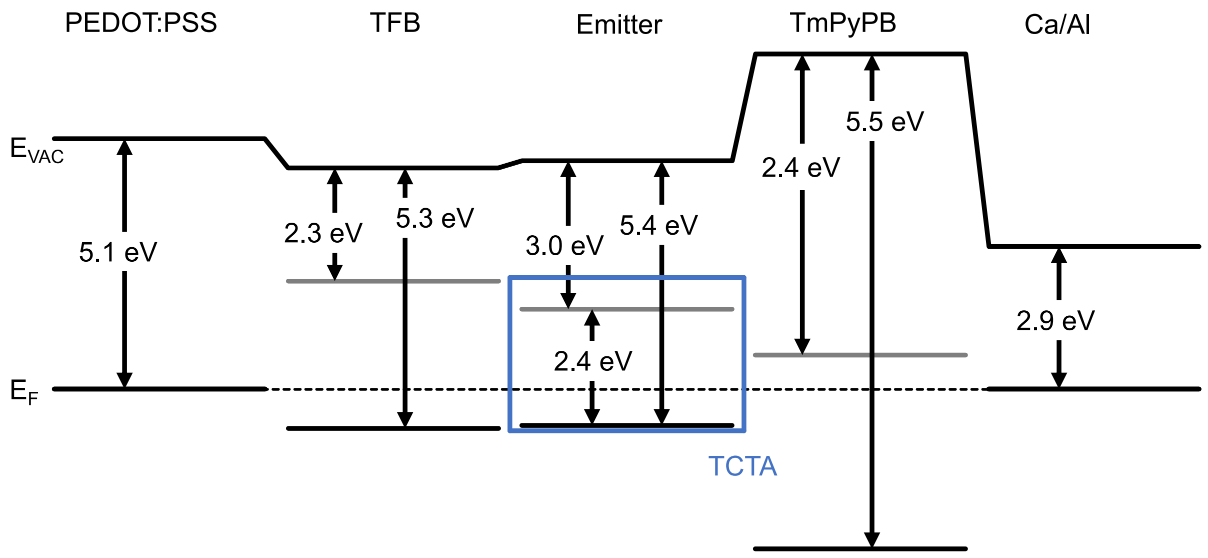

Figure S26: Energy levels of the used materials without interfacial effects.

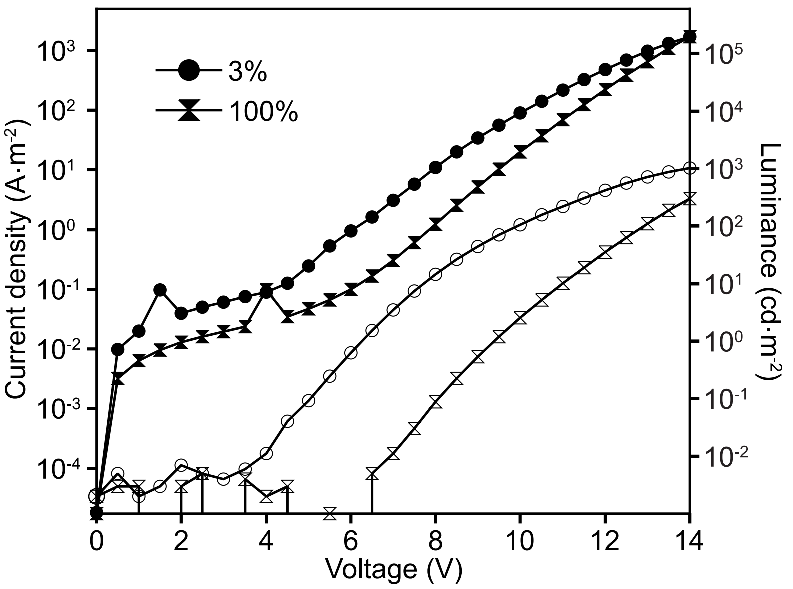

Figure S27: JVL curves for the OLEDs with blocking layers in dependence of emitter concentration. Current density shown with filled markers, luminance without fill.

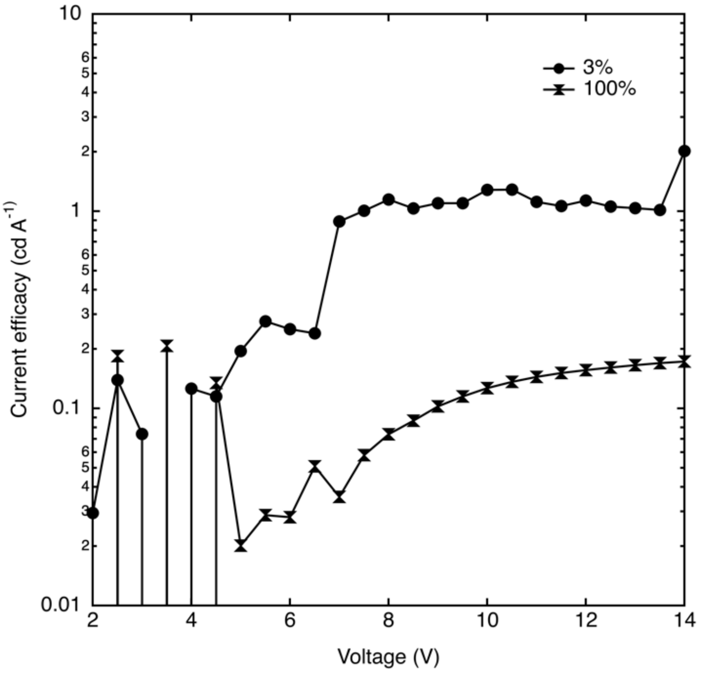

Figure S28: Current efficacies of the OLEDs with blocking layers.

For the OLEDs with blocking layers, we see the current efficacy increase at a higher voltage, matching the later turn-on from the JVL charaterizations. No decrease in efficacy is observed at the measured voltages of up to 14 V.

Table S5: Device data for all fabricated OLEDs. The luminous power efficiency is calculated by considering the OLED as an Lambertian source, that emits into the half-plane.[14–15]

Device stack	luminance maximum	turn-on	current efficacy	luminous power efficiency	CIE x	CIE y
PEDOT:PSS/**3**/Ca/Al	3458 cd m^{-2}	3 V	0.25 cd A^{-1}	0.14 lm/W	0.48	0.50
PEDOT:PSS/3% **3** in TCTA//Ca/Al	1654 cd m^{-2}	3.5 V	0.46 cd A^{-1}	0.28 lm/W	0.26	0.62
PEDOT:PSS/10% **3** in TCTA//Ca/Al	947 cd m^{-2}	3 V	0.29 cd A^{-1}	0.18 lm/W	0.28	0.62
PEDOT:PSS/20% **3** in TCTA//Ca/Al	1203 cd m^{-2}	3 V	0.13 cd A^{-1}	0.06 lm/W	0.35	0.59
PEDOT:PSS/50% **3** in TCTA//Ca/Al	2376 cd m^{-2}	3 V	0.26 cd A^{-1}	0.21 lm/W	0.43	0.53
PEDOT:PSS/80% **3** in TCTA//Ca/Al	1839 cd m^{-2}	3 V	0.24 cd A^{-1}	0.12 lm/W	0.46	0.51
PEDOT:PSS/TFB/3% **3** in TCTA/TmPyPB/Ca/Al	1584 cd m^{-2}	4 V	1.36 cd A^{-1}	0.45 lm/W	0.30	0.58
PEDOT:PSS/TFB/**3**/TmPyPB/Ca/Al	299 cd m^{-2}	7 V	0.18 cd A^{-1}	0.04 lm/W	0.49	0.48

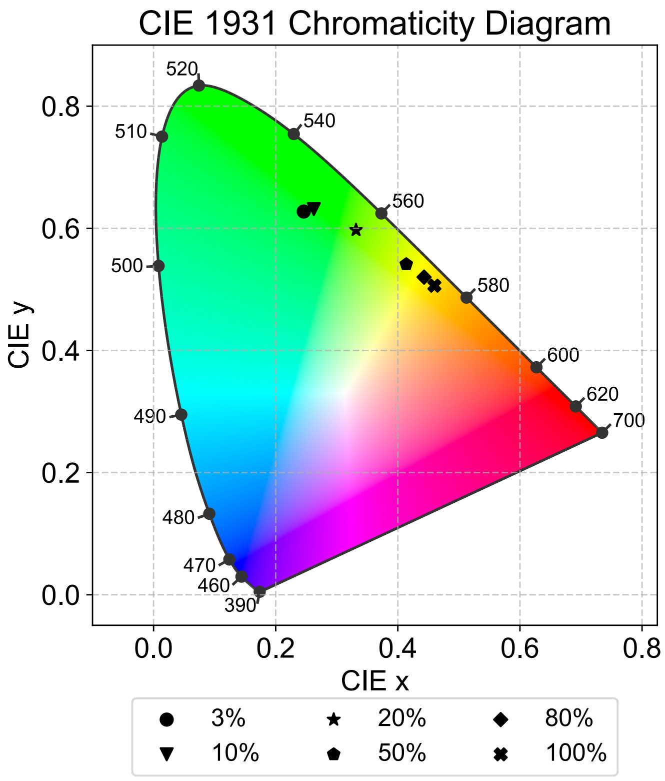

Figure S29: CIE 1931 chromaticity diagram of fabricated OLEDs with different portions of compound **3**. Upon dilution, the color shifts from orange-yellow to green.

10 References

[1] M. Götze, L. Polewski, L. Bechtella, K. Pagel, *J. Am. Soc. Mass Spectrom.* **2023**, *34*, 2403–2406.

[2] O. V. Dolomanov, L. J. Bourhis, R. J. Gildea, J. A. K. Howard, H. Puschmann, *J. Appl. Crystallogr.* **2009**, *42*, 339–341.

[3] A. L. Spek, *J. Appl. Crystallogr.* **2003**, *36*, 7–13.

[4] G. M. Sheldrick, *Acta Crystallogr., Sect. C: Struct. Chem.* **2015**, *71*, 3–8.

[5] a) U. T. Mueller-Westerhoff, M. Zhou, *J. Org. Chem.* **1994**, *59*, 4988–4992; b) S. K. Sadhukhan, C. Viala, A. Gourdon, *Synthesis* **2003**, *10*, 1521–1525; c) X. Geng, J. T. Mague, J. P. Donahue, R. A. Pascal, *J. Org. Chem.* **2016**, *81*, 3838–3847; d) J. R. Johnson, O. Grummitt, *Org. Synth.* **1943**, *23*, 92; e) G. Cheng, H. Zhang, X. Cui, *RSC Adv.* **2014**, *4*, 1849–1852; f) P. Rietsch, J. Soyka, S. Brülls, J. Er, K. Hoffmann, J. Beerhues, B. Sarkar, U. Resch-Genger, S. Eigler, *Chem. Commun.* **2019**, *55*, 10515–10518.

[6] C. Reichardt, *Chem. Rev.* **1994**, *94*, 2319–2358.

[7] a) F. Neese, *Wiley Interdiscip. Rev.: Comput. Mol. Sci.* **2022**, *12*, e1606; b) M. J. Frisch, G. W. Trucks, H. B. Schlegel, G. E. Scuseria, M. A. Robb, J. R. Cheeseman, G. Scalmani, V. Barone, G. A. Petersson, H. Nakatsuji, X. Li, M. Caricato, A. V. Marenich, J. Bloino, B. G. Janesko, R. Gomperts, B. Mennucci, H. P. Hratchian, J. V. Ortiz, A. F. Izmaylov, J. L. Sonnenberg, Williams, F. Ding, F. Lipparini, F. Egidi, J. Goings, B. Peng, A. Petrone, T. Henderson, D. Ranasinghe, V. G. Zakrzewski, J. Gao, N. Rega, G. Zheng, W. Liang, M. Hada, M. Ehara, K. Toyota, R. Fukuda, J. Hasegawa, M. Ishida, T. Nakajima, Y. Honda, O. Kitao, H. Nakai, T. Vreven, K. Throssell, J. A. Montgomery Jr., J. E. Peralta, F. Ogliaro, M. J. Bearpark, J. J. Heyd, E. N. Brothers, K. N. Kudin, V. N. Staroverov, T. A. Keith, R. Kobayashi, J. Normand, K. Raghavachari, A. P. Rendell, J. C. Burant, S. S. Iyengar, J. Tomasi, M. Cossi, J. M. Millam, M. Klene, C. Adamo, R. Cammi, J. W. Ochterski, R. L. Martin, K. Morokuma, O. Farkas, J. B. Foresman, D. J. Fox, Wallingford, CT, **2016**.

[8] a) J.-D. Chai, M. Head-Gordon, *Phys. Chem. Chem. Phys.* **2008**, *10*, 6615–6620; b) S. Grimme, J. Antony, S. Ehrlich, H. Krieg, *J. Chem. Phys.* **2010**, *132*, 154104; c) S. Grimme, S. Ehrlich, L. Goerigk, *J. Comput. Chem.* **2011**, *32*, 1456–1465; d) F. Weigend, R. Ahlrichs, *Phys. Chem. Chem. Phys.* **2005**, *7*, 3297–3305.

[9] a) A. D. Becke, *J. Chem. Phys.* **1993**, *98*, 5648–5652; b) C. Lee, W. Yang, R. G. Parr, *Phys. Rev. B* **1988**, *37*, 785–789; c) A. D. Becke, *Phys. Rev. A* **1988**, *38*, 3098–3100; d) R. Krishnan, J. S. Binkley, R. Seeger, J. A. Pople, *J. Chem. Phys.* **1980**, *72*, 650–654; e) T. Clark, J. Chandrasekhar, G. W. Spitznagel, P. von Ragué Schleyer, *J. Comput. Chem.* **1983**, *4*, 294–301.

[10] J. Tomasi, B. Menucci, R. Cammi, *Chem. Rev.* **2005**, *105*, 2999–3093.

[11] Z. Wang, *Chemistry (Basel, Switz.)* **2024**, *6*, 1692–1703.

[12] D. Geuenich, K. Hess, F. Köhler, R. Herges, *Chem. Rev.* **2005**, *105*, 3758–3772.

[13] W. J. Hehre, R. Ditchfield, J. A. Pople, *J. Chem. Phys.* **1972**, *56*, 2257–2261.

[14] S. R. Forrest, D. D. C. Bradley, M. E. Thompson, *Adv. Mater.* **2003**, *15*, 1043–1048.

[15] N. C. Greenham, R. H. Friend, D. D. C. Bradley, *Adv. Mater.* **1994**, *6*, 491–494.

11 NMR Spectra

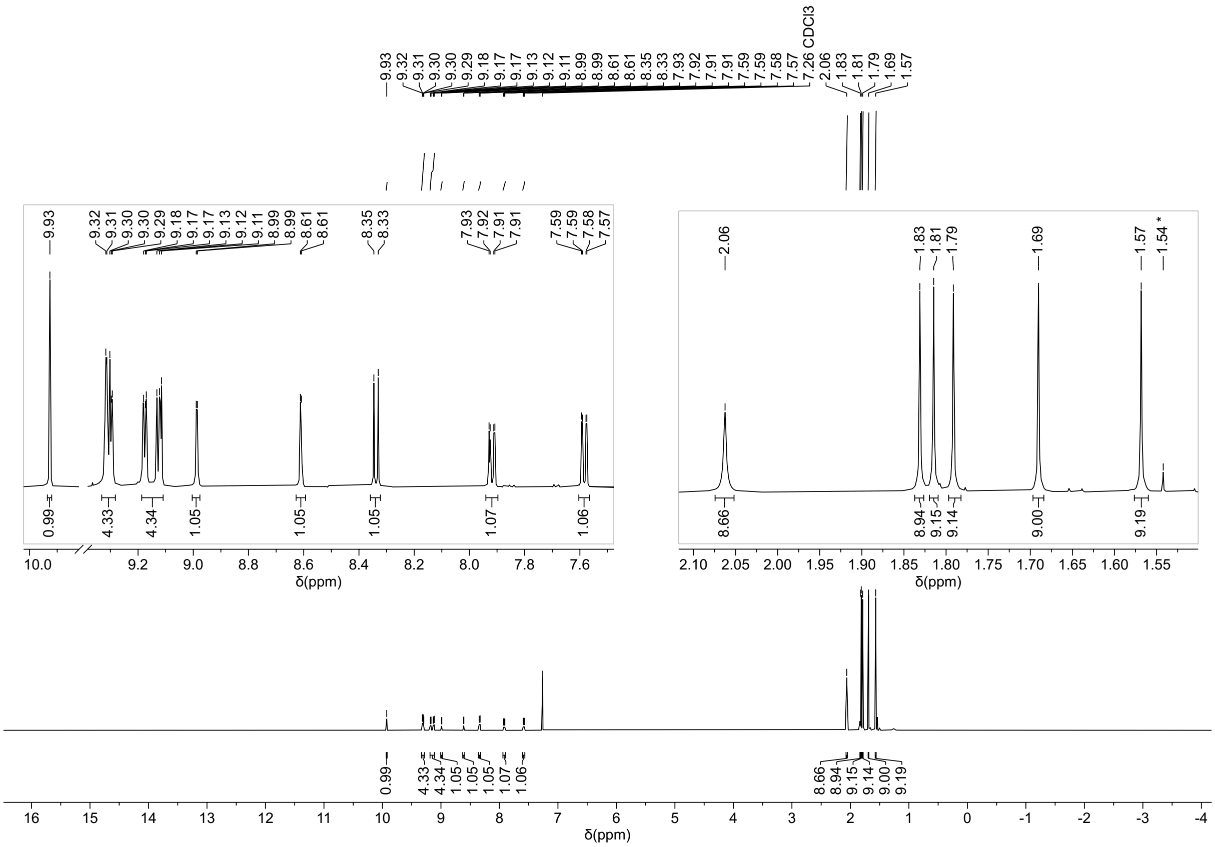

Figure S30: ^{1}H NMR spectrum (500 MHz, $CDCl_3$, rt) of compound **3**.

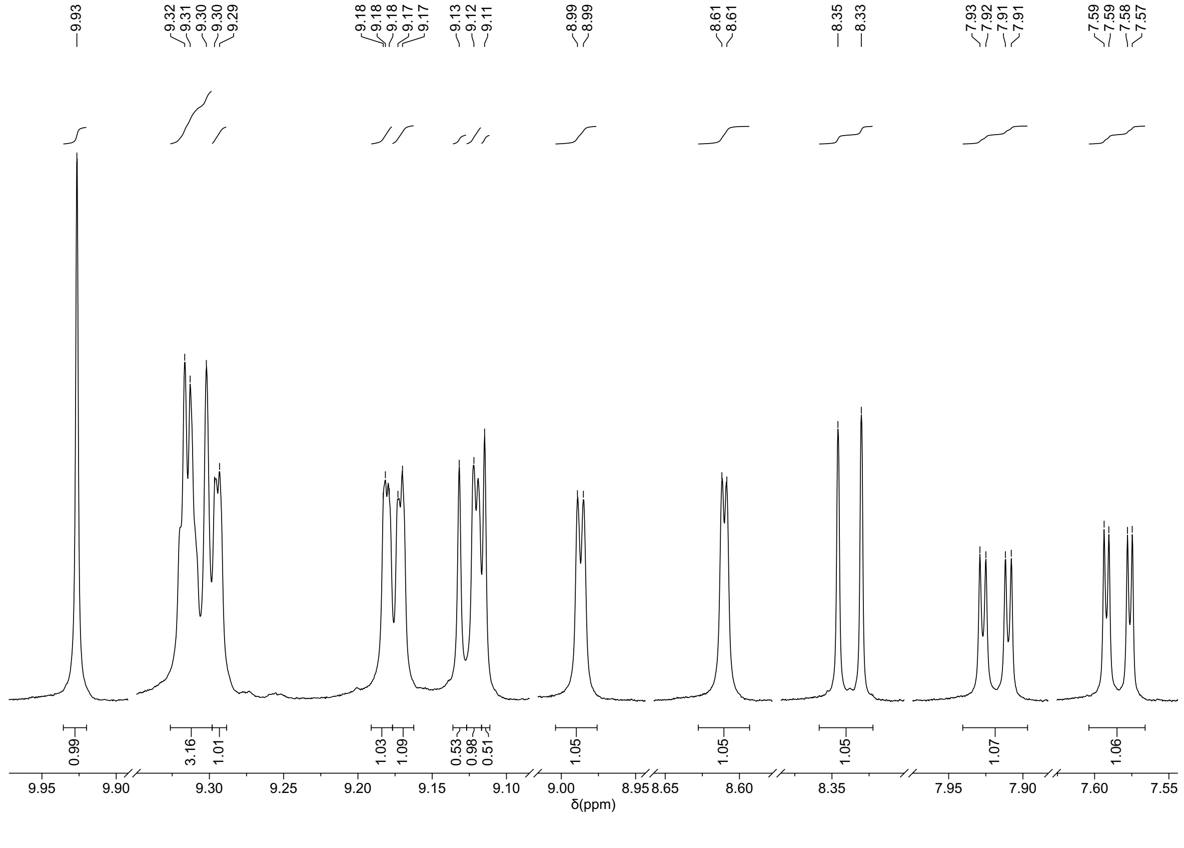

Figure S31: Expansion of the aromatic region of the ^{1}H NMR spectrum (500 MHz, $CDCl_3$, rt) of compound **3**.

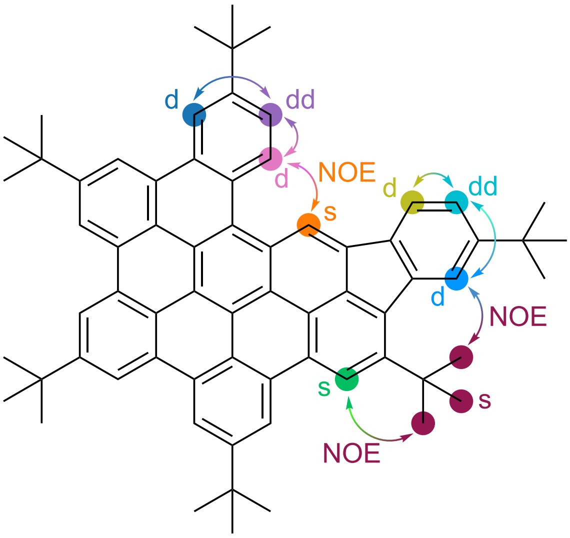

Figure S32: Schematic representation of the assigned ^{1}H couplings in the ^{1}H NMR spectrum of compound **3**.

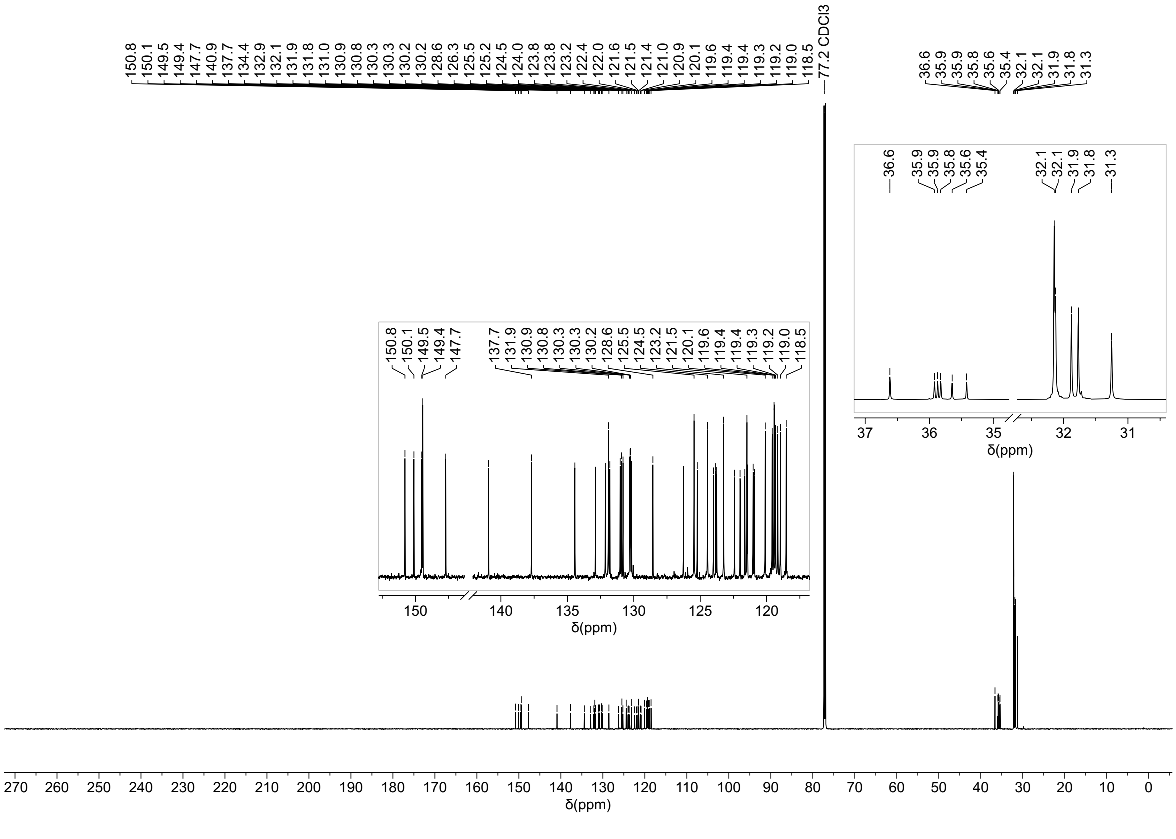

Figure S33: ^{13}C NMR spectrum (151 MHz, $CDCl_3$, rt) of compound **3**.

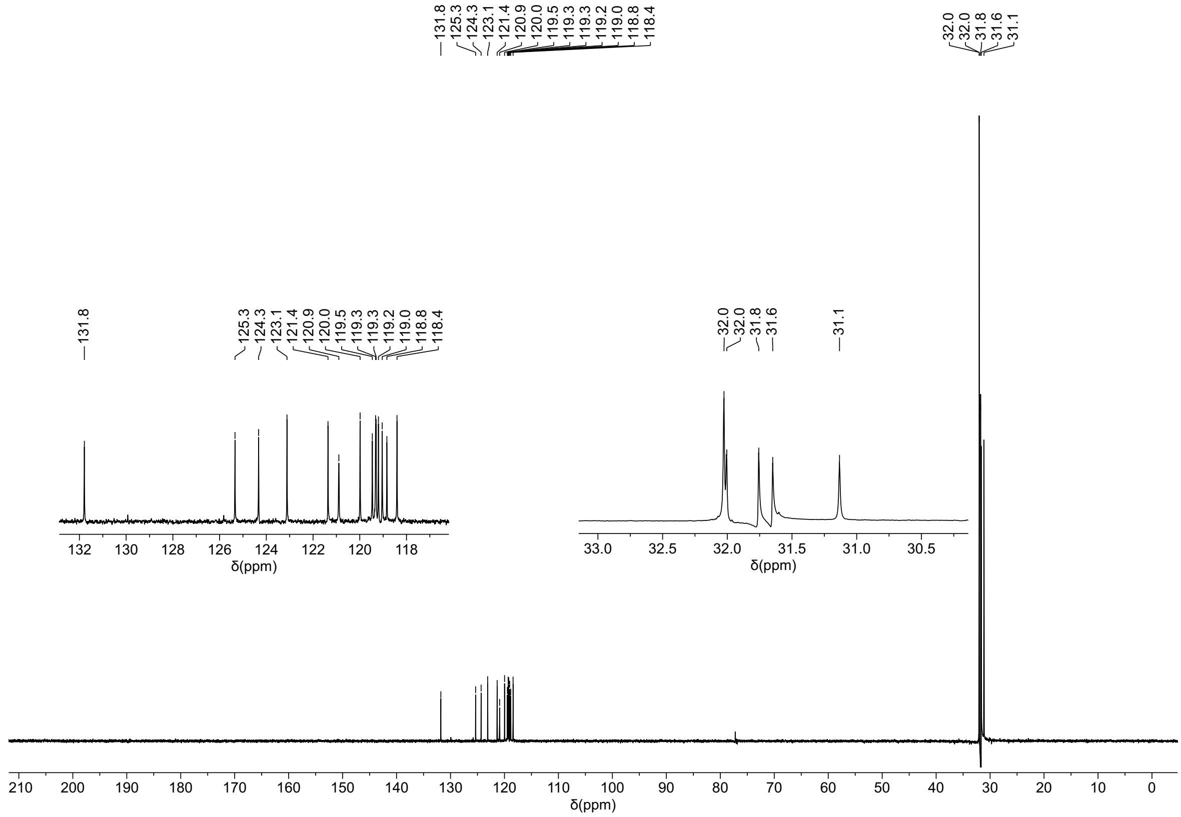

Figure S34: ^{13}C DEPT135 NMR spectrum (176 MHz, $CDCl_3$, rt) of compound **3**.

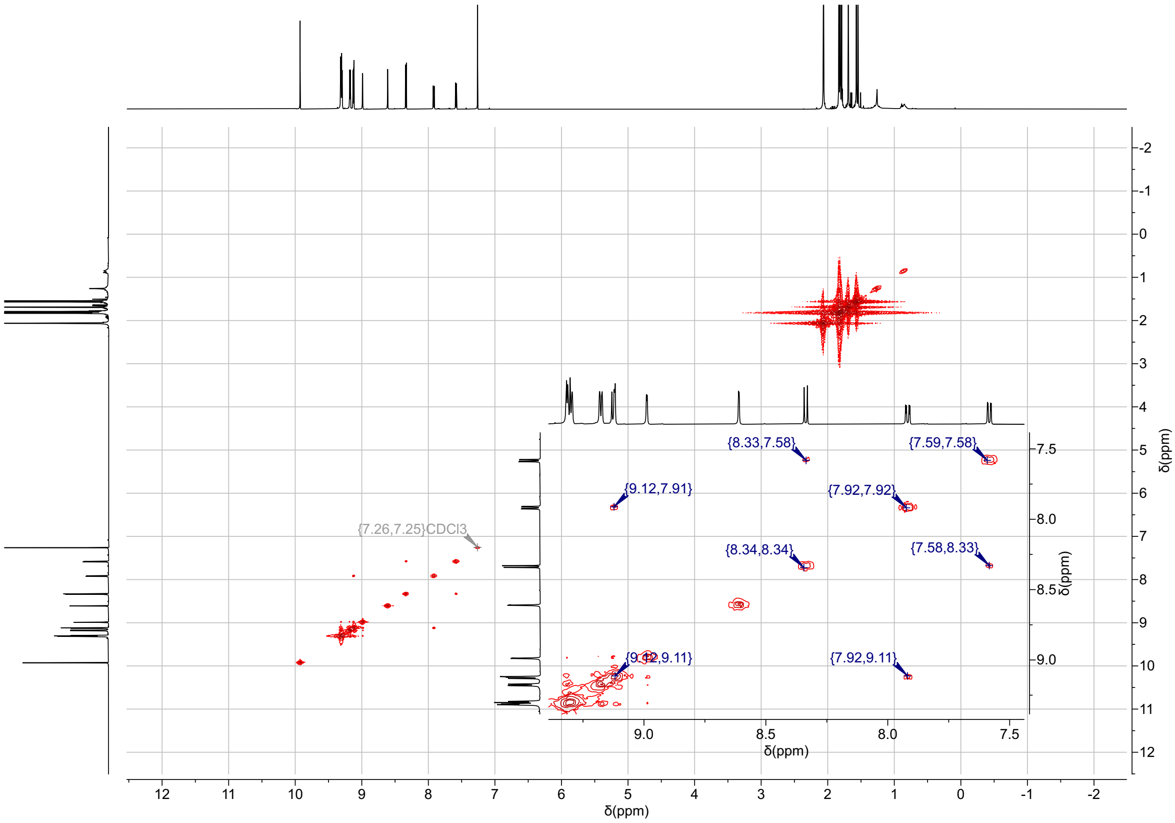

Figure S35: ^{1}H-^{1}H COSY NMR spectrum (600, 600 MHz, $CDCl_3$, rt) of compound **3**.

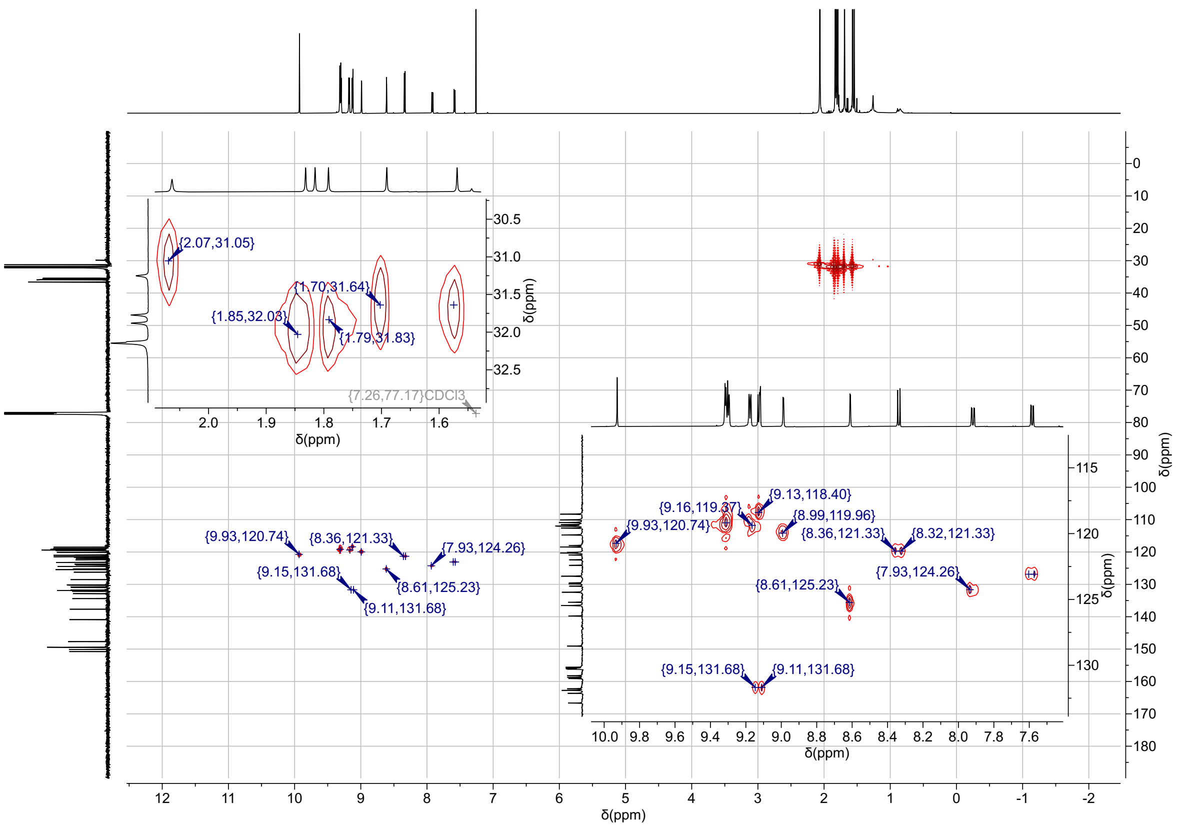

Figure S36: ^{1}H-^{13}C HMQC NMR spectrum (600, 151 MHz, $CDCl_3$, rt) of compound **3**.

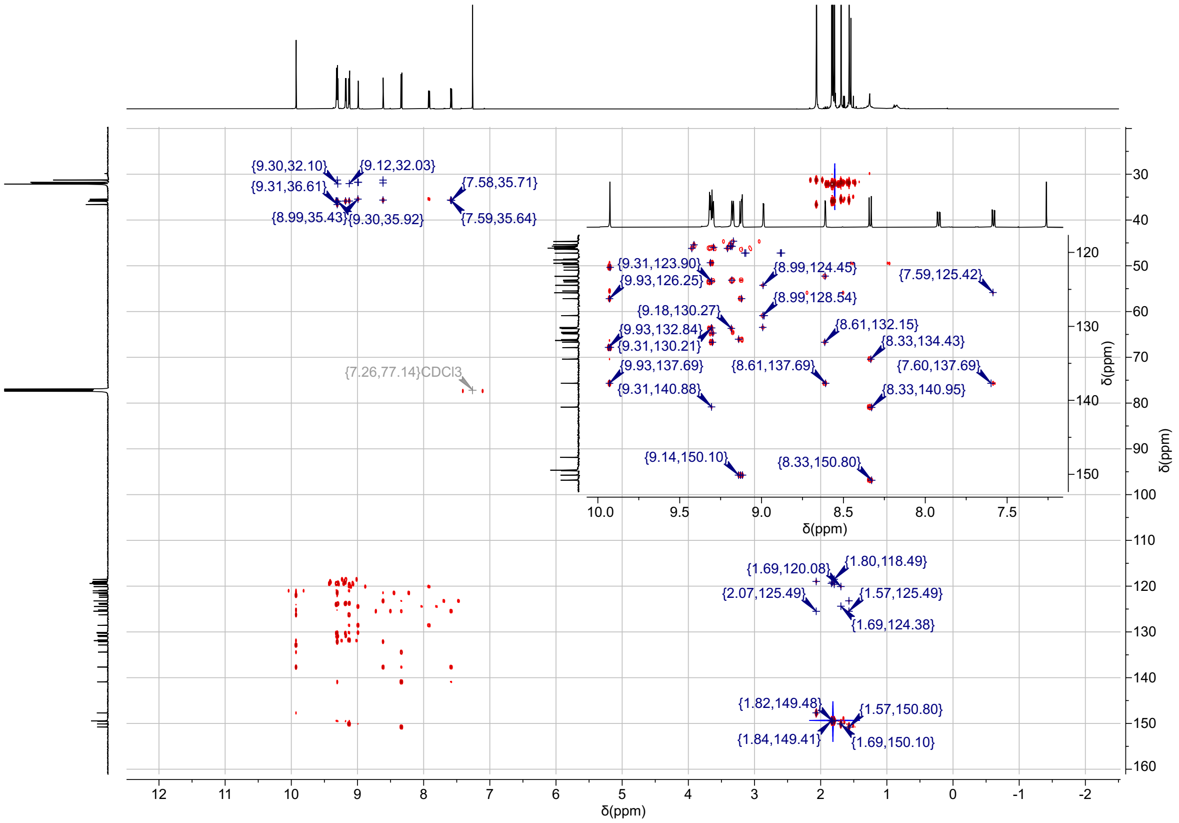

Figure S37: ^{1}H-^{13}C HMBC NMR spectrum (700, 176 MHz, $CDCl_3$, rt) of compound **3**.

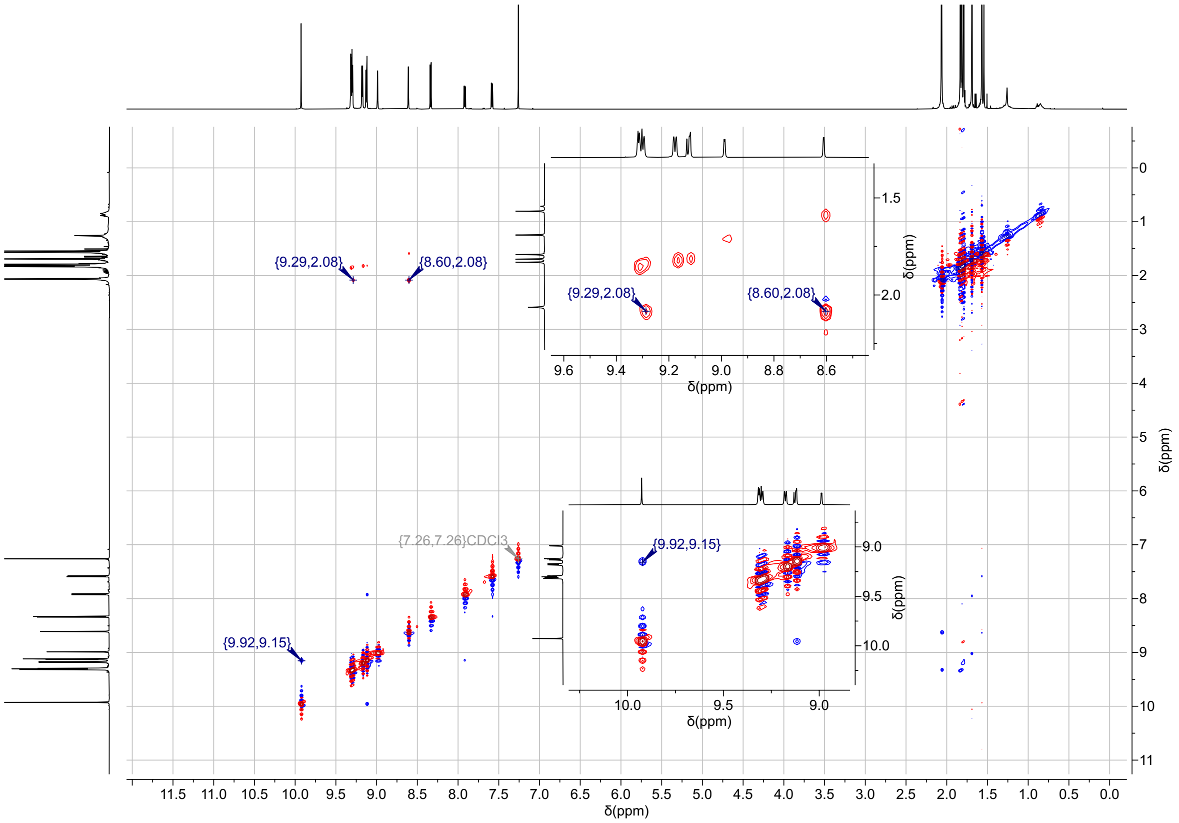

Figure S38: ^{1}H-^{1}H NOESY NMR spectrum (600, 600 MHz, $CDCl_3$, rt) of compound **3**.

12 Optimized Geometries

Compound 2

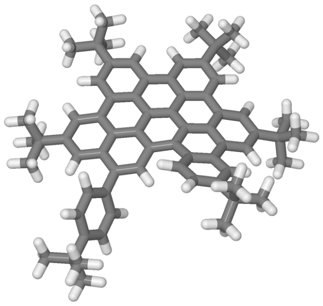

Functional/Basis set: ωB97X-D3BJ/def2-TZVP

Charge = 0, Multiplicity = 1

Electronic Energy = -2635.955813 Hartree

	Cartesian Coordinates				Cartesian Coordinates		
**Atoms**	***X***	***Y***	***Z***	**Atoms**	***X***	***Y***	***Z***
C	-0.012451	-0.114348	0.022959	C	-1.470410	-0.530926	0.218935
C	-1.848924	-1.199318	1.386901	C	-3.154941	-1.601886	1.601368
C	-4.145349	-1.350327	0.651212	C	-5.558512	-1.752318	0.883136
C	-5.926948	-3.144893	0.994259	C	-7.292707	-3.476236	1.113419
C	-8.289458	-2.443981	1.157767	C	-9.662174	-2.786397	1.152009
C	-10.621337	-1.776851	1.070123	C	-10.227009	-0.416903	1.135417
C	-8.883938	-0.078752	1.316520	C	-7.899317	-1.094031	1.178707
C	-6.510580	-0.795853	0.966409	H	-6.212956	0.232748	0.824407
C	-8.559620	1.309317	1.654623	C	-7.393967	1.657925	2.362057
C	-7.104178	2.962383	2.678521	C	-7.972436	4.006154	2.324239
C	-9.156282	3.656911	1.709958	C	-9.483139	2.327909	1.390232
C	-10.818047	1.977071	0.898293	C	-11.211644	0.623744	0.925511
C	-12.547320	0.292514	0.643152	C	-13.427029	1.295223	0.237955
C	-13.034457	2.620976	0.127729	C	-11.728916	2.940455	0.480818
H	-11.410831	3.971697	0.414364	C	-13.976548	3.725542	-0.354127
C	-15.369885	3.192324	-0.698319	H	-15.855883	2.742931	0.171713
H	-15.330475	2.447598	-1.497426	H	-15.999935	4.016536	-1.041855
C	-14.125809	4.784467	0.749717	H	-14.535768	4.338916	1.659740
H	-14.801061	5.579710	0.420873	H	-13.166538	5.241324	1.001587
C	-13.386224	4.379207	-1.613355	H	-13.268120	3.642281	-2.411900
H	-12.406980	4.819919	-1.415800	H	-14.047111	5.173548	-1.972348
H	-14.437167	1.025688	-0.027650	C	-12.984229	-1.105146	0.765473
C	-12.019803	-2.117140	0.957481	C	-12.433673	-3.457410	1.046829
C	-13.796506	-3.755329	0.963596	C	-14.757220	-2.772759	0.804995
C	-14.325763	-1.452412	0.708788	H	-15.067576	-0.673770	0.600283
C	-16.253632	-3.080828	0.732733	C	-16.973220	-2.365328	1.886928
H	-16.588665	-2.705980	2.851669	H	-18.046346	-2.574203	1.850340
H	-16.839451	-1.282842	1.835531	C	-16.544850	-4.579893	0.841512
H	-16.196514	-4.990470	1.792844	H	-16.077361	-5.141502	0.028461
H	-17.623474	-4.745515	0.782967	C	-16.811413	-2.578601	-0.608412
H	-16.678564	-1.500710	-0.721423	H	-17.881508	-2.794550	-0.677579

H	-16.306427	-3.069163	-1.444494	H	-14.112505	-4.784335	1.023856
C	-11.428702	-4.513468	1.209123	C	-10.060655	-4.171505	1.204859
C	-9.090981	-5.189904	1.275805	C	-9.505167	-6.511497	1.424653
C	-10.845966	-6.863304	1.476097	C	-11.787272	-5.850259	1.351512
H	-12.833547	-6.118418	1.376126	C	-11.315228	-8.308711	1.652934
C	-12.174944	-8.410882	2.922708	H	-13.056883	-7.769811	2.864268
H	-11.600347	-8.112523	3.803302	H	-12.515901	-9.440298	3.066146
C	-12.153279	-8.722607	0.432947	H	-13.033432	-8.087115	0.315903
H	-12.495891	-9.755674	0.542403	H	-11.562452	-8.650827	-0.483815
C	-10.145746	-9.287887	1.786684	H	-9.522949	-9.055419	2.654435
H	-9.513366	-9.286672	0.894981	H	-10.533374	-10.301312	1.917105
H	-8.760926	-7.286923	1.511763	C	-7.666292	-4.839582	1.172557
C	-6.677310	-5.807550	1.105555	C	-5.321675	-5.495693	0.971427
C	-4.967673	-4.163433	0.912879	H	-3.934273	-3.879578	0.785192
C	-4.297470	-6.627214	0.880227	C	-4.374741	-7.486074	2.152306
H	-5.363653	-7.931632	2.279169	H	-4.161247	-6.883051	3.038632
H	-3.643579	-8.298168	2.104080	C	-4.611432	-7.498655	-0.345990
H	-5.607052	-7.942694	-0.281432	H	-3.884897	-8.312389	-0.427473
H	-4.566959	-6.904662	-1.262387	C	-2.866668	-6.099965	0.742146
H	-2.579076	-5.485681	1.599297	H	-2.742325	-5.503301	-0.164883
H	-2.172422	-6.942270	0.686199	H	-6.957646	-6.850844	1.135557
H	-9.881632	4.428132	1.501273	C	-7.606267	5.448512	2.669427
C	-8.674850	6.444897	2.212595	H	-8.829483	6.400518	1.131339
H	-8.357109	7.460099	2.463152	H	-9.632454	6.264856	2.708034
C	-6.282802	5.806729	1.974989	H	-6.380444	5.719891	0.889720
H	-5.472037	5.148292	2.293317	H	-5.996342	6.835080	2.213866
C	-7.438311	5.583354	4.191015	H	-8.366290	5.322823	4.706444
H	-7.176882	6.613053	4.451643	H	-6.648555	4.931008	4.568801
H	-6.195209	3.173677	3.230281	H	-6.726244	0.881008	2.706471
C	-3.779485	-0.680598	-0.507843	C	-2.464011	-0.278558	-0.719971
H	-2.227138	0.236102	-1.642266	H	-4.532622	-0.476558	-1.261728
H	-3.417143	-2.113581	2.521659	H	-1.109245	-1.409051	2.151794
C	0.879575	-1.365566	0.051612	H	0.599301	-2.057420	-0.746910
H	0.795839	-1.896664	1.001984	H	1.927893	-1.085823	-0.087372
C	0.209957	0.604648	-1.310098	H	-0.043562	-0.034548	-2.159641
H	1.263203	0.881132	-1.403516	H	-0.382611	1.520553	-1.377344
C	0.402184	0.834540	1.158401	H	0.309799	0.356407	2.135667
H	-0.224045	1.730455	1.161139	H	1.443909	1.143187	1.031500

Compound 3

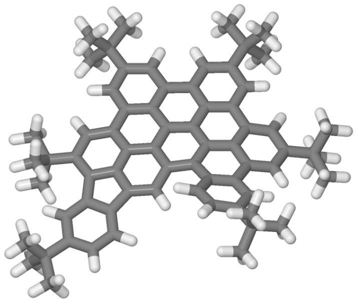

Functional/Basis set: ωB97X-D3BJ/def2-TZVP

Charge = 0, Multiplicity = 1

Electronic Energy = -2634.742813 Hartree

	Cartesian Coordinates				Cartesian Coordinates		
Atoms	X	Y	Z	Atoms	X	Y	Z
C	-0.004350	0.034210	0.000238	C	1.523106	0.027343	0.013299
C	2.225393	1.242677	0.021129	C	3.597828	1.269900	0.008676
C	4.361759	0.087659	-0.027632	C	5.825154	0.104662	-0.040304
C	6.499404	-1.009312	0.473230	C	7.893427	-0.957031	0.734713
C	8.608853	0.202922	0.445366	C	7.958945	1.259714	-0.224807
C	6.583429	1.205105	-0.530604	C	6.022516	2.220475	-1.397814
C	6.773408	3.272722	-1.793366	C	8.131183	3.378719	-1.374588
C	8.735908	2.390275	-0.623858	C	10.093440	2.547850	-0.313074
C	10.729456	3.697221	-0.777231	C	10.118987	4.698585	-1.553646
C	8.764718	4.524219	-1.874499	C	7.714255	5.226188	-2.683327
C	6.529555	4.449229	-2.634681	C	5.390367	4.848874	-3.300020
C	5.404189	6.032622	-4.033707	C	6.548579	6.819566	-4.101741
C	7.696811	6.400715	-3.417974	H	8.574880	7.022948	-3.480641
C	6.609123	8.126502	-4.895803	C	5.281072	8.449337	-5.585478
H	5.376501	9.387799	-6.137428	H	4.998056	7.670720	-6.298322
H	4.470085	8.568589	-4.862537	C	6.951768	9.284850	-3.945389
H	6.995838	10.227305	-4.499014	H	7.918337	9.136147	-3.459261
H	6.193614	9.380176	-3.163771	C	7.696795	8.013505	-5.976247
H	7.476286	7.189775	-6.659985	H	7.750233	8.938710	-6.557442
H	8.681832	7.833970	-5.540227	H	4.503394	6.331895	-4.553004
H	4.488422	4.247043	-3.255148	C	10.943976	5.902292	-2.016619
C	12.393746	5.874005	-1.513102	H	12.907948	6.768412	-1.874621
H	12.447942	5.879776	-0.421677	H	12.940840	5.005680	-1.888135
C	11.018705	5.899084	-3.554419	H	11.649020	5.070134	-3.886741
H	11.466124	6.829971	-3.915959	H	10.048955	5.773003	-4.029526
C	10.314943	7.189416	-1.452555	H	9.241011	7.245692	-1.612233
H	10.481069	7.233524	-0.373004	H	10.781666	8.071002	-1.902399
H	11.773659	3.823129	-0.539836	C	10.749282	1.483129	0.454854
C	10.010244	0.317810	0.782823	C	10.653391	-0.736985	1.452820
C	9.914109	-1.974935	1.725530	C	8.566561	-2.081464	1.335644
C	7.872982	-3.290944	1.549084	C	8.519645	-4.335673	2.190322
C	9.846293	-4.244683	2.606016	C	10.523447	-3.065442	2.353973
H	11.554665	-2.986324	2.657616	C	10.493563	-5.435031	3.315354

C	9.713095	-5.741610	4.603269	H	10.163145	-6.591939	5.123649
H	9.724863	-4.880424	5.276229	H	8.670583	-5.989258	4.393304
C	10.455261	-6.660985	2.389247	H	10.918504	-7.520822	2.881681
H	10.998240	-6.461394	1.461903	H	9.431672	-6.936163	2.126805
C	11.952914	-5.164813	3.691578	H	12.042851	-4.315825	4.374147
H	12.366662	-6.041907	4.195290	H	12.566783	-4.967489	2.808883
H	7.976167	-5.247918	2.392771	C	6.478649	-3.408817	1.097119
C	5.790076	-2.256619	0.664707	C	4.429967	-2.358930	0.325904
C	3.818552	-3.614518	0.311241	C	4.501938	-4.765558	0.661901
C	5.826322	-4.633082	1.072386	H	6.370294	-5.524088	1.353164
C	3.862859	-6.154099	0.621299	C	2.404520	-6.108349	0.157375
H	1.995835	-7.121743	0.141929	H	1.785484	-5.510449	0.831422
H	2.314938	-5.697981	-0.851699	C	4.650486	-7.044699	-0.352554
H	4.206220	-8.043377	-0.394551	H	5.693023	-7.152883	-0.046321
H	4.638554	-6.619948	-1.359545	C	3.900563	-6.773864	2.027167
H	3.353384	-6.150368	2.738905	H	3.441291	-7.766486	2.014503
H	4.923613	-6.882613	2.393047	H	2.787189	-3.688389	0.003196
C	3.675684	-1.128573	0.080465	C	2.269386	-1.129945	0.069696
H	1.756032	-2.075497	0.150663	C	11.994017	-0.589395	1.824883
C	12.717652	0.555540	1.544415	C	12.074291	1.576827	0.848442
H	12.629269	2.473949	0.609286	C	14.178933	0.733888	1.958795
C	14.726969	-0.487929	2.700992	H	15.769415	-0.308675	2.975538
H	14.170120	-0.685045	3.620768	H	14.696899	-1.385503	2.077742
C	15.039483	0.961170	0.705994	H	16.088710	1.088668	0.987485
H	14.728232	1.853518	0.159187	H	14.966623	0.107907	0.026740
C	14.293962	1.953473	2.886946	H	15.333952	2.098505	3.193247
H	13.957983	2.867719	2.393320	H	13.686551	1.813539	3.784754
H	12.483583	-1.393797	2.349565	H	5.013392	2.098441	-1.764896
H	4.097941	2.225591	0.078962	H	1.686191	2.182362	0.061298
C	-0.490021	0.773188	-1.256855	H	-1.583256	0.795125	-1.283438
H	-0.131934	1.804452	-1.279267	H	-0.133747	0.272727	-2.160995
C	-0.592705	-1.378844	-0.014987	H	-1.683292	-1.318201	-0.050159
H	-0.320723	-1.939788	0.882910	H	-0.260755	-1.943120	-0.890373
C	-0.521346	0.760713	1.251972	H	-0.180516	0.255942	2.159534
H	-0.172379	1.794569	1.290400	H	-1.615067	0.774582	1.255278

***t*-Bu-HBC**

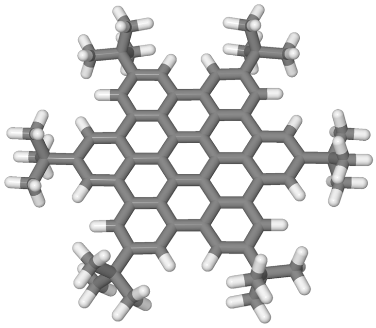

Functional/Basis set: ωB97X-D3BJ/def2-TZVP

Charge = 0, Multiplicity = 1

Electronic Energy = -2557.308511 Hartree

	Cartesian Coordinates				Cartesian Coordinates		
Atoms	*X*	*Y*	*Z*	Atoms	*X*	*Y*	*Z*
C	-0.009414	-0.048572	0.003080	C	-0.401660	-1.526764	-0.000584
C	0.589378	-2.499150	-0.001955	C	0.301473	-3.858858	-0.004631
C	-1.045569	-4.277130	-0.005641	C	-1.375125	-5.686112	-0.006297
C	-0.349106	-6.648518	-0.006362	C	-0.670086	-8.019614	-0.005965
C	-2.016818	-8.426776	-0.005851	C	-3.044043	-7.463273	-0.006341
C	-2.723271	-6.093631	-0.006658	C	-3.778192	-5.103364	-0.007737
C	-3.466476	-3.727521	-0.008044	C	-4.499481	-2.798222	-0.011010
C	-5.837524	-3.169874	-0.013047	C	-6.126578	-4.522868	-0.011793
C	-5.128043	-5.497219	-0.009001	C	-5.464383	-6.924514	-0.007587
C	-4.428681	-7.882217	-0.006774	C	-4.762169	-9.248514	-0.005804
C	-3.693738	-10.253408	-0.005833	C	-2.346300	-9.834730	-0.005727
C	-1.329107	-10.806029	-0.005738	C	0.073646	-10.382570	-0.006101
C	0.383735	-9.010976	-0.006225	C	1.737747	-8.615180	-0.007107
C	2.728557	-9.587827	-0.007321	C	2.440540	-10.947063	-0.006835
C	1.108840	-11.319493	-0.006157	H	0.867338	-12.369469	-0.005840
C	3.586159	-11.960235	-0.007131	C	3.082931	-13.406237	-0.006690
H	3.936690	-14.088485	-0.006934	H	2.483190	-13.624243	0.880835
H	2.482463	-13.624516	-0.893654	C	4.447629	-11.750645	-1.262362
H	5.271969	-12.469449	-1.279526	H	4.876471	-10.746921	-1.293759
H	3.851010	-11.889218	-2.167618	C	4.448651	-11.750226	1.247323
H	3.852803	-11.888624	2.153109	H	5.273083	-12.468938	1.263995
H	4.877408	-10.746450	1.278099	H	3.766021	-9.288612	-0.007729
C	2.072782	-7.186209	-0.007772	C	1.036540	-6.229304	-0.006582
C	1.369654	-4.863411	-0.006498	C	2.712780	-4.484873	-0.008548
C	3.740109	-5.410974	-0.010326	C	3.393284	-6.756037	-0.009624
H	4.189328	-7.485551	-0.010933	C	5.216483	-5.012455	-0.013232
C	5.408511	-3.493486	-0.013470	H	6.476551	-3.261991	-0.015573
H	4.966671	-3.030965	-0.899994	H	4.970199	-3.031128	0.874892
C	5.898408	-5.583296	1.240101	H	6.956400	-5.305862	1.254098
H	5.836448	-6.673029	1.271400	H	5.428232	-5.194093	2.146794
C	5.893290	-5.583076	-1.269435	H	5.419461	-5.193651	-2.174128
H	6.951243	-5.305728	-1.287698	H	5.831107	-6.672796	-1.300688
H	2.963857	-3.437080	-0.009341	C	-1.673188	-12.158679	-0.005902

C	-2.988992	-12.585056	-0.006034	C	-3.980955	-11.612604	-0.005993
H	-5.010700	-11.937555	-0.006310	C	-3.381247	-14.063239	-0.006346
C	-2.160732	-14.987915	-0.006421	H	-2.493135	-16.028950	-0.006708
H	-1.540469	-14.836297	-0.893624	H	-1.540673	-14.836713	0.880995
C	-4.214943	-14.367199	1.248327	H	-4.503679	-15.422163	1.263503
H	-5.127640	-13.768620	1.280715	H	-3.641243	-14.154214	2.153977
C	-4.214677	-14.366699	-1.261327	H	-3.640782	-14.153324	-2.166762
H	-4.503378	-15.421665	-1.277006	H	-5.127380	-13.768127	-1.293669
H	-0.891160	-12.899661	-0.006083	C	-6.104827	-9.626341	-0.005054
C	-7.132310	-8.699409	-0.005333	C	-6.785778	-7.354882	-0.006701
H	-7.581940	-6.625569	-0.006799	C	-8.608775	-9.097990	-0.004090
C	-8.800851	-10.616968	-0.002881	H	-9.868918	-10.848409	-0.002029
H	-8.361367	-11.080090	-0.890273	H	-8.360232	-11.078838	0.884598
C	-9.287316	-8.526276	1.250688	H	-10.345191	-8.804022	1.267919
H	-9.225558	-7.436511	1.280665	H	-8.814465	-8.914625	2.156373
C	-9.289071	-8.528090	-1.258745	H	-8.817473	-8.917716	-2.164535
H	-10.346955	-8.805908	-1.274085	H	-9.227433	-7.438370	-1.290371
H	-6.356362	-10.674063	-0.003967	H	-7.159536	-4.829189	-0.013281
C	-6.920871	-2.090313	-0.016513	C	-8.332358	-2.683763	-0.018317
H	-9.067047	-1.874750	-0.020855	H	-8.510460	-3.297598	-0.905135
H	-8.513788	-3.295023	0.869615	C	-6.768673	-1.214878	1.237351
H	-7.538214	-0.437649	1.250728	H	-5.794069	-0.723574	1.269730
H	-6.871665	-1.817176	2.143605	C	-6.763939	-1.218306	-1.272172
H	-6.863478	-1.823074	-2.177168	H	-7.533392	-0.441089	-1.290605
H	-5.789202	-0.727112	-1.302185	H	-4.265035	-1.744272	-0.012252
C	-2.061899	-3.305182	-0.005455	C	-1.717952	-1.953469	-0.002605
H	-2.500201	-1.212663	-0.001320	H	1.619234	-2.174661	-0.000357
C	-1.229957	0.876062	0.004255	H	-0.897636	1.917106	0.006905
H	-1.849240	0.726697	-0.884050	H	-1.850623	0.722604	0.890896
C	0.823125	0.252348	1.259254	H	1.111705	1.307303	1.277374
H	1.735862	-0.346199	1.290830	H	0.248731	0.036928	2.163887
C	0.825052	0.258047	-1.250429	H	0.251988	0.046824	-2.156890
H	1.113722	1.313056	-1.263284	H	1.737787	-0.340437	-1.283376
